# Accuracy of gait and posture classification using movement sensors in individuals with mobility impairment after stroke

**DOI:** 10.3389/fphys.2022.933987

**Published:** 2022-09-26

**Authors:** Johannes Pohl, Alain Ryser, Janne Marieke Veerbeek, Geert Verheyden, Julia Elisabeth Vogt, Andreas Rüdiger Luft, Chris Awai Easthope

**Affiliations:** ^1^ Department of Neurology, University of Zurich and University Hospital Zurich, Zurich, Switzerland; ^2^ Department of Rehabilitation Sciences, KU Leuven—University of Leuven, Leuven, Belgium; ^3^ Department of Computer Science, ETH Zurich, Zurich, Switzerland; ^4^ Neurocenter, Luzerner Kantonsspital, Lucerne, Switzerland; ^5^ Cereneo, Center for Neurology and Rehabilitation, Vitznau, Switzerland; ^6^ Cereneo Foundation, Center for Interdisciplinary Research (CEFIR), Vitznau, Switzerland

**Keywords:** physical activity, body posture, gait, real-life, movement sensor, stroke

## Abstract

**Background:** Stroke leads to motor impairment which reduces physical activity, negatively affects social participation, and increases the risk of secondary cardiovascular events. Continuous monitoring of physical activity with motion sensors is promising to allow the prescription of tailored treatments in a timely manner. Accurate classification of gait activities and body posture is necessary to extract actionable information for outcome measures from unstructured motion data. We here develop and validate a solution for various sensor configurations specifically for a stroke population.

**Methods:** Video and movement sensor data (locations: wrists, ankles, and chest) were collected from fourteen stroke survivors with motor impairment who performed real-life activities in their home environment. Video data were labeled for five classes of gait and body postures and three classes of transitions that served as ground truth. We trained support vector machine (SVM), logistic regression (LR), and k-nearest neighbor (kNN) models to identify gait bouts only or gait and posture. Model performance was assessed by the nested leave-one-subject-out protocol and compared across five different sensor placement configurations.

**Results:** Our method achieved very good performance when predicting real-life gait versus non-gait (*Gait classification*) with an accuracy between 85% and 93% across sensor configurations, using SVM and LR modeling. On the much more challenging task of discriminating between the body postures lying, sitting, and standing as well as walking, and stair ascent/descent (*Gait and postures classification*), our method achieves accuracies between 80% and 86% with at least one ankle and wrist sensor attached unilaterally. The *Gait and postures classification* performance between SVM and LR was equivalent but superior to kNN.

**Conclusion:** This work presents a comparison of performance when classifying Gait and body postures in post-stroke individuals with different sensor configurations, which provide options for subsequent outcome evaluation. We achieved accurate classification of gait and postures performed in a real-life setting by individuals with a wide range of motor impairments due to stroke. This validated classifier will hopefully prove a useful resource to researchers and clinicians in the increasingly important field of digital health in the form of remote movement monitoring using motion sensors.

## Introduction

Motor impairment after stroke reduces the level of daily physical activities and mobility and negatively affects social participation (de Rooij et al., 2021). In comparison to healthy controls, stroke survivors show significantly lower energy expenditure, more time spent sedentary, and less stepping activity in the acute (Kramer et al., 2013; Moore et al., 2013), subacute and chronic phases of motor recovery after stroke (Rand et al., 2009; English et al., 2016; Ezeugwu and Manns, 2017). Continuous inactivity and sedentary behavior are associated with an increased risk of recurrent stroke events (Wendel-Vos, 2004). Consequently, monitoring physical activities and implementing secondary prevention strategies are one of the main targets of health care providers.

According to the World Health Organization, daily physical activity measures should be structured in four components: frequency, intensity, time, and type (FITT) (Cavill et al., 2006). The quantitative and qualitative analysis of physical activities requires a hypothesis-driven set of outcome metrics considered relevant for the studied population. Regarding monitoring motor recovery and movement behavior in individuals with stroke, a minimal set of relevant outcome metrics which addresses upper and lower limb performance was proposed by van Meulen et al. (2016). Sitting, standing, and walking were considered types of physical activities of which duration, intensity, and quality of movement should be analyzed (van Meulen et al., 2016). Movement sensors are increasingly used as an unobtrusive approach to continuously monitor real-life physical activity in various populations with and without neurologic diseases (Block et al., 2016; Del Din et al., 2016). Especially neurologic populations returning from in-patient rehabilitation are a prime target for monitoring daily physical activity (Prajapati et al., 2011). Gains in motor function that are achieved during in-patient therapy are frequently not successfully translated into home routines (Thilarajah et al., 2016, 2018). Many patients with persistent motor impairment consult outpatient rehabilitation interventions that support them in maintaining and successfully integrating new motor capabilities into their home routine (Ostwald et al., 2009). Early identification of a lack of transfer of in-patient motor gains to home routine would allow for active referral of at-risk patients to avoid long-term complications or functional decline.

Movement sensors deployed in home environments generate large-scale unstructured and unlabeled time series data (Ehatisham-ul-Haq and Azam, 2020). The extraction of metrics corresponding to the most relevant outcomes proposed by van Meulen is not trivial (van Meulen et al., 2016). Thus, a significant body of research has emerged dedicated to the automated classification of physical activity types (e.g., walking and standing) and to calculating intensity and movement quality metrics once the activity type is determined (e.g., step count, energy expenditure, and stride symmetry in a walking period). The accurate classification of physical activity is paramount to analysis, as it provides metrics of frequency and duration and identifies the data sequences that enable subsequent evaluation of activity-specific metrics. Although neurologic populations are a primary target group for remote monitoring in home environments, most activity classification algorithms have been developed and validated for healthy populations within laboratory conditions (Gyllensten and Bonomi, 2011; O’Brien et al., 2017). Healthy populations arguably demonstrate a higher signal-to-noise ratio, characterized by greater movement speeds and lower movement variability (Balasubramanian et al., 2009; Stergiou and Decker, 2011; Atrsaei et al., 2020; Atrsaei et al., 2021). Applying these algorithms to neurologic populations in home environments substantially reduces activity classification accuracy (Jayaraman et al., 2018). To our knowledge, only a few studies have attempted to develop and validate physical activity classification algorithms specifically for neurologic populations such as stroke (Leuenberger et al., 2014; Massé et al., 2015; Derungs et al., 2020). In these studies, movement tasks were predefined and performed in standard clinical environments. To confidently apply these classification algorithms in home environments, the ecological validity must be demonstrated in real-life settings.

Accuracy of activity classification depends heavily on the sensor technology (e.g., modalities, dimensionality, sampling rate), data processing (preprocessing and classification algorithm), the number and location of sensors, and the characteristics of targeted physical activity types (set of activities, patient-specific movement characteristics, environment) (Clark et al., 2017; Clark et al., 2018; Allahbakhshi et al., 2019; Rast and Labruyère, 2020; Wu et al., 2021; Boukhennoufa et al., 2022) Although all mentioned components are important, the optimal number and location of sensors are essential to accurately detect diverse types of physical activity. This is especially relevant as a low number of sensors contributes strongly to participant comfort and adherence in remote settings and reduces the technological complexity of the recording setup. In healthy individuals, a minimal sensor setup for basic activities such as sitting, standing, and walking requires two sensors, ideally placed on the waist and ankle (Allahbakhshi et al., 2019). However, additional sensors are desirable to increase the number of detectable activities and provide a basis for movement intensity and quality, such as real-world gait analysis (Renggli et al., 2020; Werner et al., 2021).

Accordingly, our study has the following objectives: firstly, we compare the performance of three frequently used machine learning algorithms to classify gait and posture in real-world environments for subjects with motor impairment after stroke. Secondly, we compare the performance across five typical sensor configurations.

## Materials and methods

### Subjects

Subjects enrolled in a prospective observational study at the University Hospital Zurich were invited to participate if individuals met the following inclusion criteria: mono- or hemiparesis after stroke, ability to walk independently (with or without walking aid) with a Functional Ambulation Categories (FAC) score ≥3/5, living at home, aged above 18 years. Subjects were informed regarding the goal and procedure of the study and provided written informed consent for their participation. Ethical clearance to conduct the study was provided by the cantonal ethics committee Zurich Switzerland (BASEC No. Req-2020-00947).

### Measurement device

**FIGURE 1:**
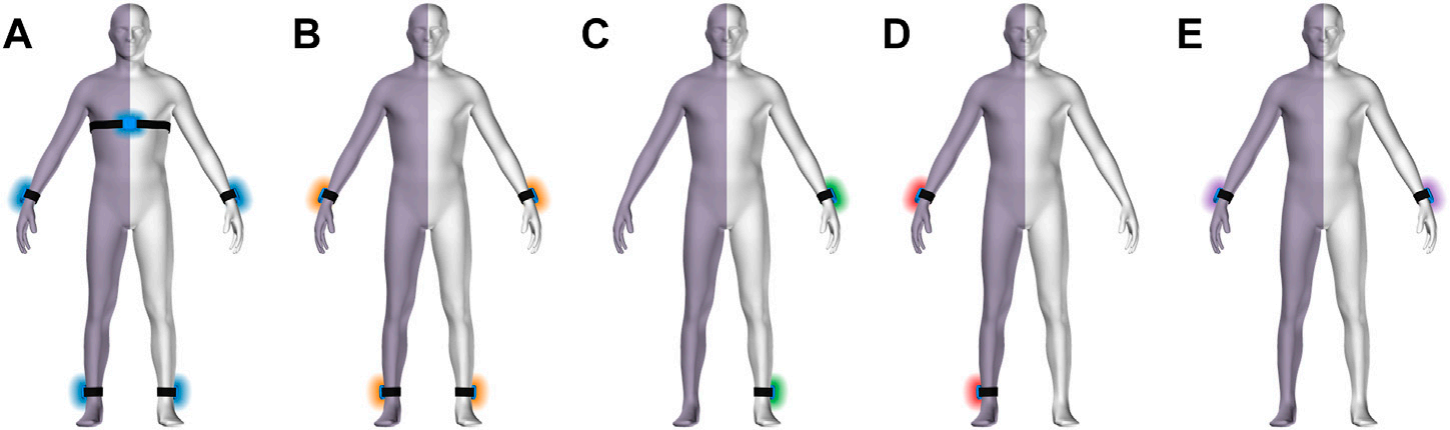
Body scheme with hemi-paretic side symbolized by violet shade. Sensor configurations **(A–D)** with sensors locations: all sensors **(A)**, no chest sensor, bilateral wrist and ankle **(B)**, unilateral wrist and ankle on the non-affected side **(C)**, unilateral wrist and ankle on the affected side **(D)**, and wrist sensors only **(E)**.

Five movement sensors developed for research purposes (https://zurichmove.com/) were attached with elastic straps, one on the dorsal side of the wrists, at the lateral malleolus of the ankles, and on the chest below the sternum (Figure 1A). These inertial measurement units (IMU) include a 3-axis accelerometer, a 3-axis gyroscope, a 3-axis digital compass, an altimeter (10 cm resolution), a storage capacity of 4 GB, and a rechargeable battery that enables recordings of up to 72 h. Sensor configuration was adjusted to a sampling frequency of 50 Hz, and synchronization between modules was achieved *via* a radio frequency syncing protocol.

### Procedure

A semi-structured protocol containing routine daily life activities was developed following the recommendations for standardized validation procedures for activity and posture classification (Lindemann et al., 2014). The subjects were visited at home, where they were asked to perform tasks that they would typically perform throughout a regular day, beginning with getting up (e.g., morning routine, grooming, dressing). The activity plot started with an open question, e.g., “What do you typically do first after getting up in the morning? Please proceed with this activity if I were not present.” Individuals subsequently performed their routine activities triggered by similar questions involving kitchen work, desk work, setting up a table, eating, preparing coffee, and cleaning up. These activities involved various uninstructed upper limb activities, body postures, walking, and stair ascent/descent. Participants were also asked to perform their typical leisure activities, which included reading, writing, watching TV, exercising, or playing a musical instrument. Thereby the observers remained reserved, leaving the order of actions or way how to perform a task to the individual’s preference. Exceptionally for stair walking activities, instructions were given to perform stair ascent and descent using both step-over-step and step-by-step patterns if the participant was capable to perform both patterns. Individuals with muscle weakness due to hemiplegia typically adopt the step-by-step pattern. For safety reasons, participants were supervised or assisted if they had difficulties performing a step-over-step pattern. Physical activities were recorded using a conventional video camera (GoPro Hero7, GoPro. Inc., San Mateo, United States) with 30 frames per second (fps). Synchronization between video and sensor data was obtained by video-recording an instructed knocking and turning sequence of the unaffected wrists, which provided salient signals in both systems.

### Labeling and segmentation

Video data were recorded with a framerate of 30 fps, whereas time-series from the IMU were collected with 50 fps. A single experimenter labeled videos in a frame-by-frame manner, and the labels were subsequently resampled to match the frequency of the synchronized IMUs. For quality assessment, a random sample of 33.3% of data was labeled by a second experimenter. The labeling procedure and quality assessment were conducted using Labelbox online software (https://labelbox.com).

Labeling criteria were defined for start-to-end conditions of three body postures (lying down, sitting, standing) and two gait types (walking and stair ascent/descent). Additionally, we annotated three transition labels between two corresponding posture or gait types (lying down/sit, sit/stand, stand/walk) without specifying directionality (e.g., sit-to-stand or stand-to-sit). This labeling scheme resulted in a discrete label for every frame of the recording. Labeling criteria are presented in detail in the Supplementary Table S1.

### Preprocessing

To remove noisy and irrelevant frequencies from the collected IMU time-series, we incorporated the preprocessing steps suggested in the work of Moncada-Torres et al. (2014). The proposed collection of filter operations for IMU data suppress noise and extract frequency bands corresponding to subject posture and activity and can be found in full detail in the original work (Moncada-Torres et al., 2014). This process results in four types of time-series, namely the three triaxial signals posture acceleration, activity acceleration, and gyroscope, as well as the filtered barometric signal, containing data related to posture/orientation, movement, position, and altitude, respectively. Feature characteristics are presented in the Supplementary Table S2. These signals were computed for each sensor location and subsequently split into windows of 128 time samples with an overlap of 64 samples as described in previous work (Bonomi et al., 2009; Preece et al., 2009). For subsequent sliding-window-based analysis, the majority label was assigned for each time window.

### Feature extraction

To fit a model to the data, we extracted a series of features from each of the four time-series. For each window of the posture and activity acceleration, gyroscope, and barometric signals, we extracted the features suggested by Moncada-Torres et al. (2014). This amounted to 134 features per sensor, resulting in feature vectors with 670 dimensions considering all sensors. To handle intra-subject variability, we standardized each feature per subject to zero mean and unit variance over each window, which was previously shown to increase separability, repeatability, and clustering (Krausz et al., 2019). This entails the advantage that each feature’s magnitude is relative to its magnitudes in other windows of the same subject, effectively negating the effect of varying magnitude across subjects.

More specifically, for a subject with n windows and feature vectors $x^{\left(1\right)},...,x^{\left(n\right)}\in R^{d}$ , we compute ${\tilde{x}}^{\left(1\right)},...,{\tilde{x}}^{\left(n\right)}\in R^{d}$ such that ${\tilde{x}}_{j}^{\left(i\right)}∼N\left(0,1\right)$ , i.e., we standardize each feature with ${\tilde{x}}_{j}^{\left(i\right)}=\frac{x_{j}^{\left(i\right)}-\mu_{j}}{\sigma_{j}}$ where $\mu_{j}=\frac{1}{n}\overset{n}{\underset{i=1}{\sum }}x_{j}^{\left(i\right)}$ and $\sigma_{j}^{2}=\frac{1}{n}\overset{n}{\underset{i=1}{\sum }}{\left(\mu_{j}-x_{j}^{\left(i\right)}\right)}^{2}$ .

### Classification tasks and performance analysis

The labeled dataset described in the previous sections was used to solve two classification tasks. First, we aggregated labels for *walking* and *stair ascent/descent* as “*gait*,” whereas *lying, sitting,* and *standing* were aggregated into the unified label “*no gait*.” These aggregated labels were used to solve a binary classification task, namely *Gait classification*. Our second classification task was appointed *Gait and posture classification* included all five gait activity and body posture labels to solve a multiclass classification task*.* Note that for both tasks, we removed all windows labeled with transition classes from the training set but not from the validation set to evaluate our method’s generalization to real-life data where transitions are present. Validation, including transition done as follows: Let *A* be a specific posture or activity and *B* the posture or activity following *A*. The time window containing the transition from class *A* to *B* is then correctly classified if a model predicts either *A* or *B.* Evaluation of our method was done in a nested leave-one-subject-out fashion. This protocol estimates how well model scores generalize to unseen data by defining two loops: the outer and inner loop. In every outer iteration, the outer loop splits the dataset into an outer training and test set, where the test set consists of the data of one patient, and the training set contains the rest of the data. The inner loop then takes the outer training set and performs leave-one-subject-out cross-validation. In every inner iteration, we defined an inner test set over the data of one patient of the outer training set and assigned the rest to the inner training set. Each model is fitted on this training set multiple times with different hyperparameter configurations, subsequently computing scores on the inner test set. After we computed scores for each individual, we aggregated scores across all inner splits and retrained the configuration with the best scores on the whole outer training set. Finally, we recomputed all scores on the outer test set with the retrained models. We do this for every patient and report the results by aggregating scores across all outer splits. We further compared cross-validation performance between experiments when including and excluding transitions from validation sets for both classification tasks. Model performance was evaluated by computing sensitivity, specificity, positive predictive value (PPV), and accuracy in a one vs. rest fashion (Ballabio et al., 2018). As these metrics are sensitive to class imbalance, we also evaluated balanced accuracy, defined as the arithmetic mean between sensitivity and specificity (Ballabio et al., 2018). A potential relationship between classification performance and functional motor impairment (Berg Balance Score; 10-m walking speed) was analyzed for the full sensor setup, the unilateral setup (non-affected side), and the wrists-only setup (see Figures 1A, C, E). Normality was determined by the Shapiro-Wilks test, and Pearson or Spearman correlation was applied accordingly.

### Classification models

To solve the two classification tasks, we explored three different classifiers, namely support vector machine (SVM), logistic regression (LR), and k-nearest neighbor (kNN), for comparison*.* Each of these methods required us to set a combination of several hyperparameters, which is non-trivial, as different sensor setups (Figures 1A–E) and classification tasks require different settings. Consequently, we performed a cross-validated grid search (Sunkad and Soujanya, 2016; Rabbi et al., 2021). To this end, we defined a list of possible hyperparameters for each model (Table 1) and sensor configuration within a classification task. To evaluate the generalizability of the cross-validated grid-search, we computed the nested leave-one-subject-out cross-validation scores, where we performed the inner loop using each hyperparameter combination. To decide which model to retrain in the inner loop, we used the configuration achieving the best balanced accuracy as it accounts for class imbalance, thus ensuring that models that consistently predict the same class are not selected. Optimal hyperparameters regarding classification tasks and sensor configuration are presented in the Supplementary Table S3.

**TABLE 1:** Cross-validated grid search hyperparameter combinations.

Model	Parameter	Values
SVM	C	0.01, 0.1, 1, 10
	Kernel	Radial basis function
	Class weight	Balanced
LR	C	0.001, 0.01, 0.1
	l1 ratio	0.01, 0.1
	Penalty	Elastic Net
	Intercept	True
	Class weight	Balanced
kNN	n	1, 2, 4, 8, 12, 16, 20, 24, 28, 32
	Weights	Uniform, distance

kNN, k-Nearest Neighbor; LR, logistic regression; SVM, Support Vector Machine.

To explore misclassification rates across classes for the optimal classification setup, we mapped true classes versus predicted classes by confusion matrices. All experiments were conducted using Python (v3.7, Python Software Foundation, https://www.python.org/), and model implementations were imported from the Scikit-Learn library (https://scikit-learn.org/).

## Results

Real-life data of fourteen individuals with mild-to-moderate mobility impairments were visited and recorded in their home environment. Participants’ characteristics and scores of clinical gait and balance assessments can be seen in Table 2. The agreement rate between labelers was 93.5%. The total volume of labeled video data amounted to 379 min containing an imbalance of classes ranging from 4.8% (lying) to 32.8% (standing) of averaged cumulative duration (Table 3). The average bout duration of activity from start to end was shortest for transitions (range 2.7–4.7 s) and longest for lying.

**TABLE 2:** Participants’ demographic data (*N* = 14).

Characteristics	Median	(Range)
Age (years)	73	(50–91)
Gender (f, %)	50.0	
Weight (kg)	75.5	(53–95)
Height (cm)	169.5	(152–186)
FAC (/5)	4	(3–5)
Walking aid (%)	21.0	
BBS (/56)	51	(35–56)
TUG (s)	15.0	(10.8–68.6)
10MWT (m/s)	0.8	(0.3–1.4)

BBS, Berg Balance Scale; FAC, Functional Ambulation Categories; TUG, Timed-Up and Go; 10 MWT, 10 Meter Walk Test; gender and walking are relative frequencies.

**TABLE 3:** Descriptive activity labels.

Class	Type	Duration	
		Bout (s)	Total (%)
Posture	Lying	30.3	4.8
	Sitting	20.8	20.2
	Standing	12.2	32.8
Gait	Walk	19.8	19.5
	Stairs	11.9	6.5
Transition	Ly-sit	4.0	1.2
	Sit-stand	2.7	4.5
	Stand-walk	4.7	10.5

Average duration in seconds of start-to-end of class; frequency of class relative to total recording length in %.

### Gait classification

**FIGURE 2:**
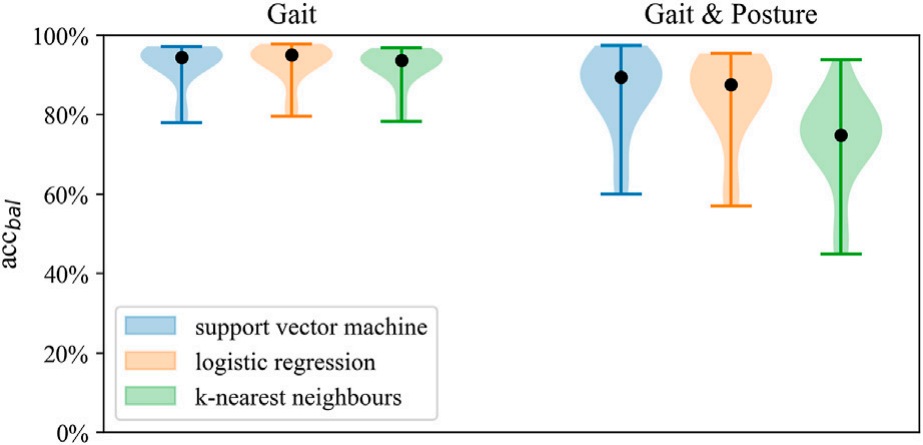
Distribution of balanced accuracy across models for *Gait classification* and *Gait and posture classification* on the full sensor setup.

**FIGURE 3:**
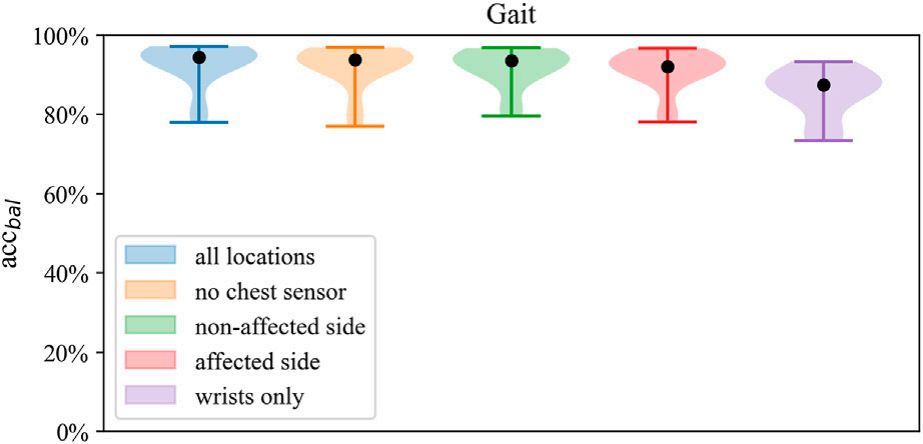
Distributions of balanced accuracy of the SVM in *Gait classification* across sensor setups.

**FIGURE 4:**
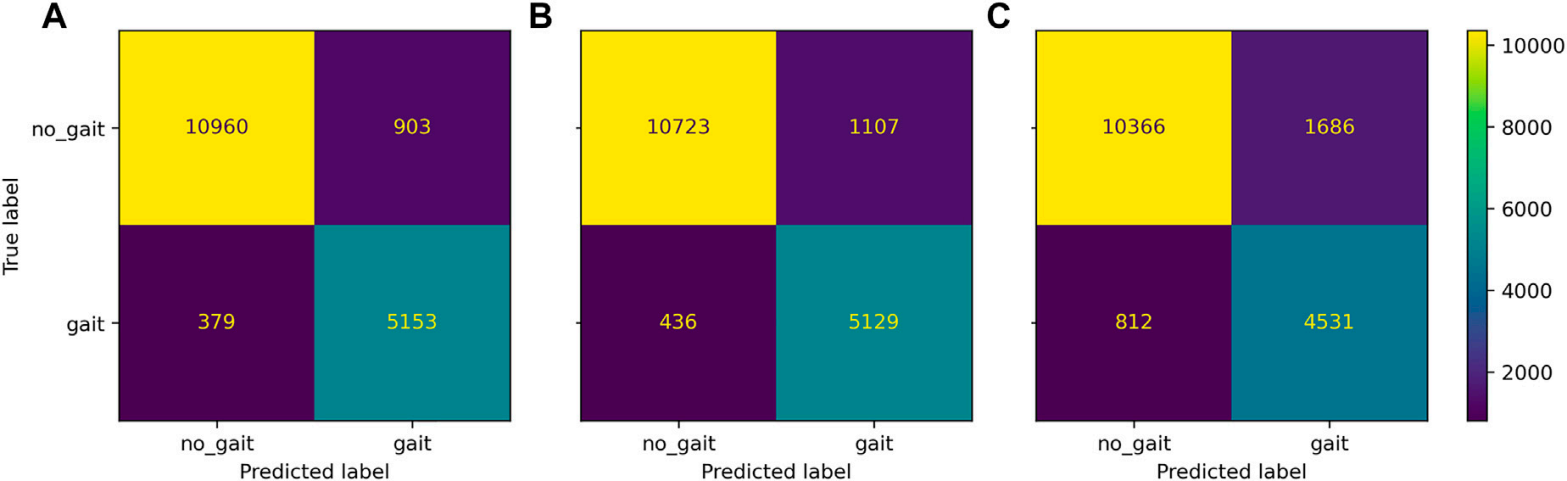
Confusion matrix of *Gait* classification task for the three district sensor setups: bilateral setup all sensors, **(A)**, unilateral setup non-affected ankle and wrist sensor, **(B)**, wrists-only setup **(C)**.

Gait classification performed consistently well across the classification models (Figure 2), achieving accuracy above 90% in most configurations (Figure 3). Across sensor setups (Figures 1A–E), SVM achieved slightly superior performance, improving in balanced accuracy over kNN by 0.9%–3.5% and over LR by 0.2%–1.0% (Table 4). Misclassifications were low using all sensors (6%) and the unilateral configuration (7%) but were increased to 15% using only wrist sensors (Figure 4).

**TABLE 4:** Model performance for *gait* classification across sensor configurations (setup).

Setup	Model	Sens	Spec	Acc	Acc_bal_	PPV
All	SVM	90.7 ± 7.5	94.5 ± 4.2	93.3 ± 4.3	92.6 ± 5.5	85.6 ± 15.3
	LR	92.1 ± 6.1	93.7 ± 4.8	93.1 ± 4.6	92.9 ± 5.1	84.5 ± 16
	kNN	89.5 ± 6.3	94.4 ± 4.6	92.7 ± 4.3	91.9 ± 4.9	85.5 ± 15.4
No chest	SVM	89.8 ± 7.3	94.2 ± 4.6	92.7 ± 4.5	92.0 ± 5.5	85.0 ± 15.7
	LR	91.4 ± 6.5	93.5 ± 4.9	92.7 ± 4.7	92.5 ± 5.3	84.1 ± 15.9
	kNN	88.5 ± 6.9	94.2 ± 4.5	92.3 ± 4.3	91.3 ± 5.3	85.0 ± 15.5
Non affected	SVM	89.2 ± 7.2	94.2 ± 4	92.5 ± 3.9	91.7 ± 5	84.8 ± 15.1
	LR	90.6 ± 6.2	93.1 ± 5.1	92.0 ± 4.8	91.8 ± 4.9	83.0 ± 16.8
	kNN	86.9 ± 6.6	94.0 ± 4.2	91.5 ± 4	90.4 ± 4.9	84.4 ± 15
Affected	SVM	89.5 ± 7.1	92.6 ± 4	91.6 ± 4.1	91.0 ± 5.1	82.0 ± 15.2
	LR	90.4 ± 6.8	92.1 ± 4.9	91.3 ± 4.6	91.3 ± 5.2	81.3 ± 16.4
	kNN	86.4 ± 8.1	93.7 ± 4.5	91.2 ± 4.3	90.0 ± 5.4	83.9 ± 15.5
Wrists-only	SVM	79.9 ± 9.4	90.6 ± 4.3	87.1 ± 4.5	85.3 ± 6.1	76.7 ± 16.9
	LR	82.8 ± 8	86.6 ± 5.4	84.9 ± 5.1	84.7 ± 5.5	71.8 ± 17.1
	kNN	80.0 ± 9.1	83.8 ± 4.6	82.4 ± 5.1	81.9 ± 5.9	67.5 ± 16.4

Mean and standard deviations of performance measures in %. Acc_bal_, balanced accuracy; kNN, k-Nearest Neighbor; LR, logistic regression; PPV, positive predictive value.

Across classifiers, performance measures marginally decreased when reducing the number of sensors, ranging from best balanced accuracy of 92.6% (all sensors, SVM) to 63.9% balanced accuracy (wrists-only, kNN). Compared to the wrists-only setup, balanced accuracy improved by 5%–8% with a unilateral setup of one wrist and ankle sensor on the affected side. Minor differences of 0.3%–0.8% within models were found between the affected and non-affected unilateral setup and between the full setup and the bilateral setup of wrists and ankles (Table 4). Misclassification frequencies for a bilateral unilateral and wrists-only setup are presented in Figure 4. The correlation between gait classification performance and functional impairment was non-significant (*p* > 0.05) across sensor configurations. *Gait* classification performance across classifiers on individual participant level is presented for the all sensors setup (Supplementary Figure S1; Supplementary Table S7), unilateral non-affected (Supplementary Figure S2; Supplementary Table S8), and the wrists-only setup (Supplementary Figure S3; Supplementary Table S9) in the supplement.

**FIGURE 5:**
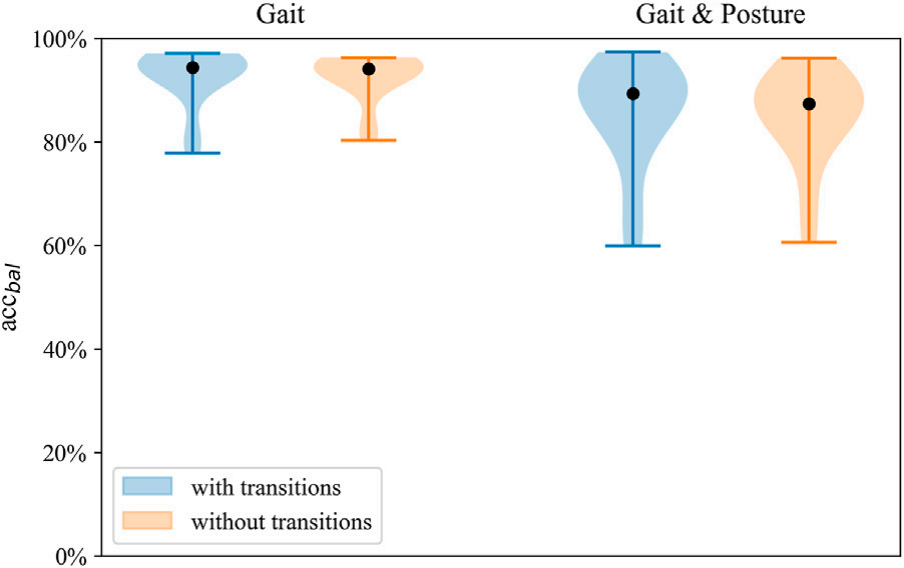
Distribution of balanced accuracy of the SVM with and without transitions on the full sensor setup for both classification tasks.

Model performance of the gait classification task was robust across validation tasks, including and excluding transitions (Figure 5). Gait classification performance validated with and without transitions can be seen in the online Supplementary Table S4.

### Gait and posture classification

**FIGURE 6:**
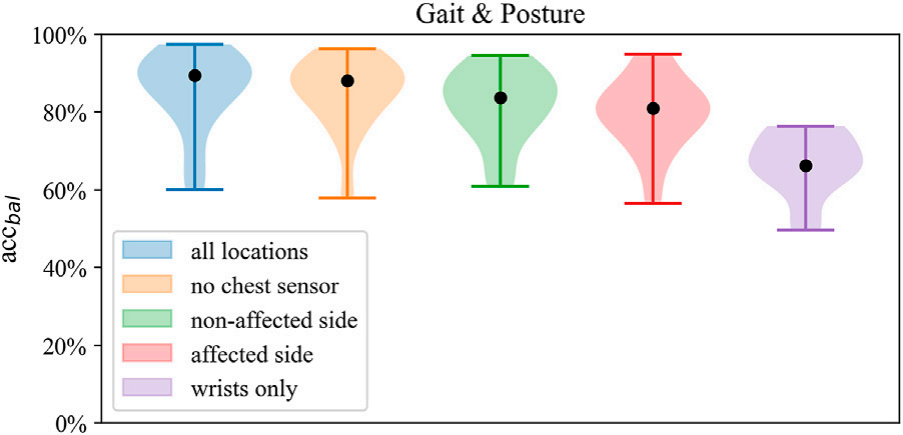
Distribution of balanced accuracy of the SVM in *Gait and posture classification* across sensor configurations.

Best performance on Gait and Posture Classification was obtained by SVM using the full sensor setup resulting in balanced accuracy (SD) of 84.7% (9.2) followed by logistic regression with 82.9% (7.6). Performance varied considerably between models where SVM and LR showed higher balanced accuracy by 10%–14% compared to kNN across sensor configurations. Balanced accuracy scores by the SVM classifier are presented in Table 5 and Figure 6, whereas a comparison of performance between classifiers is presented in Figure 3. Detailed *Gait and posture* performance measures of all classifiers are presented in the online Supplementary Table S5.

**TABLE 5:** *Gait and Posture* classification performance across sensor configurations (setups).

	Lying			Sitting			Standing			Walking			Stairs			Overall	
Setup	Sens	Spec	PPV	Sens	Spec	PPV	Sens	Spec	PPV	Sens	Spec	PPV	Sens	Spec	PPV	Acc	Acc_bal_
All	93 ± 15.3	99 ± 0.6	86 ± 14.5	87 ± 11.7	96 ± 3.3	86 ± 10.6	87 ± 7.1	92 ± 5.2	84 ± 11.8	84 ± 12.7	95 ± 3.5	82 ± 16.3	75 ± 5.3	98 ± 2.5	85 ± 4.4	85 ± 7.7	86 ± 10.2
No chest	96 ± 7.5	99 ± 0.7	86 ± 13.1	84 ± 11.5	94 ± 3.2	82 ± 12.8	85 ± 7.4	91 ± 6	84 ± 11.6	85 ± 12.1	95 ± 3.8	82 ± 16.7	73 ± 5.1	98 ± 2.2	83 ± 4.5	84 ± 8.4	85 ± 9.5
Non-aff.	92 ± 15.1	99 ± 0.8	85 ± 10	79 ± 10.6	92 ± 2.7	76 ± 12.6	81 ± 5.9	90 ± 5	81 ± 10.3	84 ± 14.2	95 ± 3.5	81 ± 16	69 ± 4.7	98 ± 3.2	79 ± 4.5	81 ± 7.1	82 ± 9.3
Aff.	86 ± 26.8	98 ± 1.9	77 ± 20.2	78 ± 11.4	91 ± 5.5	73 ± 15.7	81 ± 8.6	90 ± 5.2	82 ± 10.9	83 ± 10.5	95 ± 4	81 ± 16.7	67 ± 5.7	98 ± 1.3	75 ± 3.9	79 ± 7.5	79 ± 9.4
Wrists	69 ± 24.9	94 ± 5	49 ± 23.3	64 ± 10.3	87 ± 6.3	63 ± 19.9	66 ± 11.8	86 ± 5.1	71 ± 9.9	71 ± 14.8	91 ± 4.9	69 ± 19.7	61 ± 6.2	98 ± 1.9	68 ± 4	66 ± 9.3	67 ± 8

Mean and standard deviations of performance measures in %. Acc, accuracy; Acc_bal_, balanced accuracy; Sens, sensitivity; Spec, specificity; PPV, positive predictive value.

Across sensor configurations, around 80% of balanced accuracy was obtained when at least one wrist and one ankle sensor were included, which was reduced by 12%–15% when using only wrist sensor data. Compared to the affected side, the unilateral configuration of the non-affected side improved balanced accuracy by 0.1%–1.8%, whereas compared to both unilateral setups, the bilateral setup (no chest) improved balanced accuracy by 2%–5%. Within both bilateral sensor configurations, there was no improvement in performance by the chest sensor.

Regarding the prediction of Gait and posture classes, lying was detected with the highest sensitivity (93%) and specificity (99.0%), whereas stair walking showed the lowest sensitivity (74.8%) using the full sensor configuration. When classifying lying, sitting, and standing, positive predictive values ranged from 80.8% to 78.8%. Classification of walking exhibited a positive predictive value of 81.1%, whereas, for stair walking, PPV was 68.7%. Robust performance was found in nested leave-one-subject-out cross-validation of the *Gait and posture classification* task irrespective of the inclusion or exclusion of transitions in the validation data set (Figure 5). Detailed performance across classifiers, excluding transitions, is shown in the Supplementary Table S6.

**FIGURE 7:**
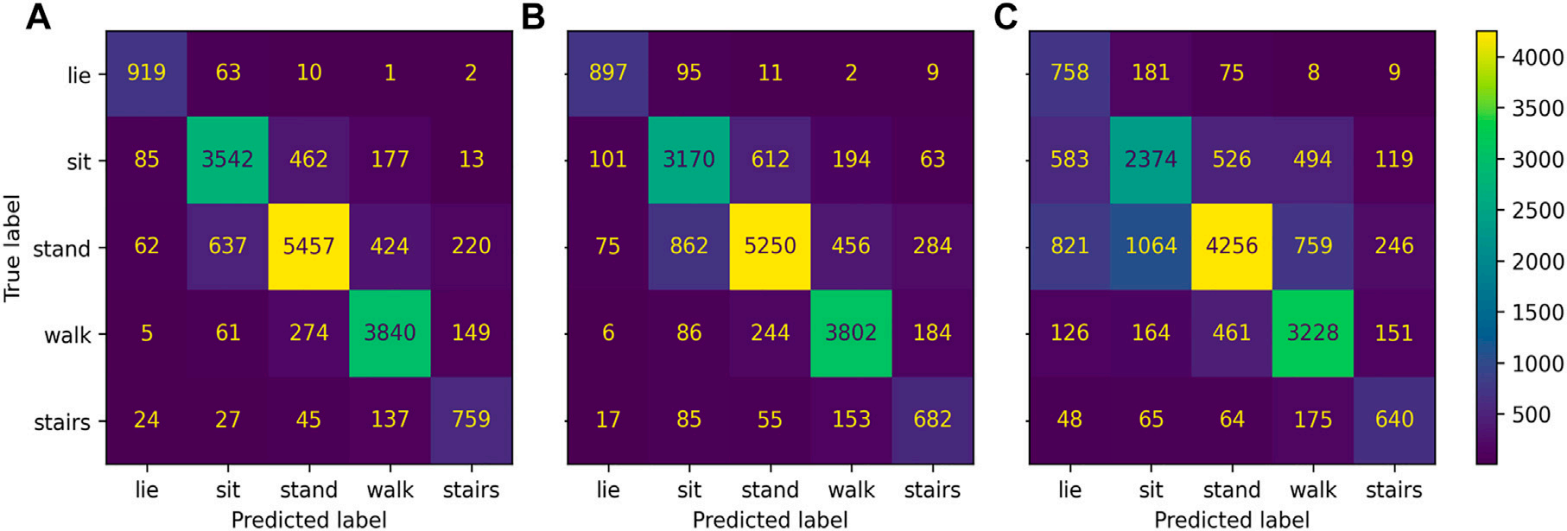
Confusion matrix *Gait and Posture* classification task for the three district sensor setups: bilateral setup all sensors, **(A)**, unilateral setup (non-affected ankle and wrist sensor, **(B)**, wrists-only setup **(C)**.

Misclassification frequencies are displayed in Figure 7 for a bilateral (A, all sensors), unilateral (B, non-affected), and wrist-only setup (C). Regarding the full sensor configuration, misclassifications were highest by predicting standing instead of sitting (11.0%), sitting instead of standing (9.4%), and walking instead of stair walking (13.8%) but were low, when predicting walking instead of standing (6.2%) and vice versa (6.2%). Using the unilateral setup, misclassification increased by 3.0%–3.8% between standing and walking and by 1.1%–2.4% between walking and stair walking. By using the wrists-only setup, misclassifications 11.2%–17.6% across the classed lying, sitting, standing, and walking. Spearman correlations between classification performance and impairment scores were not statistically significant for the bilateral and unilateral setup (*p* > 0.05). Using the wrists-only sensors, a significant negative correlation was found between walking speed and overall *Gait and posture* classification performance (*ρ* = −0.54 to −0.76; *p* < 0.05) and classification of walking (*ρ* = −0.53 to −0.60; *p* < 0.05). Relationships between classification performance walking and walking speed are presented across sensor setups in the Supplementary Figure S1. *Gait and posture* classification performance across classifiers on individual participant level is presented for the all sensors setup (Supplementary Figure S4; Supplementary Table S10), unilateral non-affected (Supplementary Figure S5; Supplementary Table S11), and the wrists-only setup (Supplementary Figure S6; Supplementary Table S12) in the supplement.

## Discussion

This study provides insight into real-life Gait and Postures Classification accuracy in individuals with varying motor impairment due to stroke. We compared the performance of state-of-the-art machine learning algorithms on Gait Classification and Gait and Posture Classification including transitions across five sensor configurations. All sensor configurations, including at least one ankle sensor, resulted in high accuracy for both classification tasks. The level of accuracy was consistent in the nested cross-validation independent of the exclusion of transitions, indicating that this algorithm is sufficiently robust to handle comprehensive real-life data. In the following section, we discuss the main performance characteristics of each classification task and address their practical implications for research and clinical rehabilitation settings.

### Gait and non-gait classification

Human movement behavior is complex and shows high variability across age groups and health conditions (Stergiou and Decker, 2011; König et al., 2016; Stergiou et al., 2016). Despite this, we applied a severe simplification by dividing basic activities into the two classes of walking and non-walking. This distinction is especially relevant for activity classification for two reasons: Firstly, it creates categories to quantify physical activity by whole-body movements during *Gait* as opposed to low activity/inactivity in static conditions during *Non-Gait* bouts. Secondly, it enables the extraction of walking periods for qualitative spatiotemporal gait analysis. The distinction between walking and non-walking is one of the main contributors to estimating energy expenditure, widely considered a benchmark outcome for monitoring physical activities (Kristoffersson and Lindén, 2020; Kristoffersson and Lindén, 2022; Boukhennoufa et al., 2022).

In our sample, a very high proportion, above 90% of recordings, was correctly classified as walking and non-walking using at least one ankle and wrist sensor. Similar results were shown by Leuenberger et al. (2014), who achieved equivalent sensitivity and specificity in detecting walking activities with all sensors compared to ankle and chest sensors (Moncada-Torres et al., 2014). Sensor location showed a clear effect on performance measures. In particular, omitting ankle sensors reduced accuracy, which plicates previous studies’ conclusions (Giggins et al., 2017). Real-life gait classification accuracy relies on arm swing patterns which might have led to a significant reduction in accuracy and precision in our sample when using only wrist sensors. However, a still acceptable level with a PPV of 73% was maintained compared to a PPV of 80% recently achieved in free-living conditions of elderly individuals using wrist sensors (Soltani et al., 2020). The overall lower accuracy and precision for the wrist-only setup could be due to very slow walking speeds (median 0.8 m/s; range 0.3–1.4), using a walking aid, or having severe arm paresis in three participants, affecting arm swing patterns. It is important to note that our classification task for *Gait* activities included overground walking, stair ascent, and descent where arm swing was absent due to asymmetric stepping pattern, holding on to rail and walker, or even a very low cadence. These pathognomonic movement patterns with hemiparesis have likely reduced the classification performance of all sensor configurations—especially the wrist-only configuration. In line with this hypothesis, Fulk et al. (2014) found step detection accuracy by wearing one wrist sensor to be reduced when post-stroke individuals had higher levels of motor impairment measured by the Berg Balance Scale (>49/56 points) and gait speed (<0.7 m/s) measured by the 2 Minute Walk Test. Using only a single sensor for gait classification has been reported as a viable option in healthy populations (Lee and Kwan, 2018); performance significantly degrades when applied in neurological populations (Capela et al., 2016) such as stroke.

The classification of *Gait* and *Non-Gait* is only considered a first step in enabling a more detailed analysis of motion, which often requires additional sensors. Detailed analysis of walking motion in individuals with stroke and spinal cord injury using a single sensor resulted in considerable measurement errors of walking distance and step count when using wrist or arm-mounted accelerometers (Jayaraman et al., 2018; Compagnat et al., 2019). Measurement errors were smaller when locating the sensor at the hips, at the ankles, or when applying multiple sensors (Werner et al., 2021).

Our sample is small but comprises a wide range of motor and mobility impairments ranging from dependency on supervision for overground walking and dependency on walking aids to normal walking ability. With most PPVs above 80%, the proportion of false-positive detected walking remains small, which is essential when investigating gait bout frequency throughout the day or creating summary intensity and quality metrics from gait sequences.

### Gait and posture classification

The full sensor configuration achieved the best classification performance across all Gait and Posture classes was achieved using the full sensor configuration. The classes lying, sitting, standing, and walking were classified with a good overall accuracy of 85%. The performance was relatively robust and remained independent from functional impairment when using at least one ankle and wrist sensor. When reducing the sensor configuration to wrists-only, the overall accuracy declined to about 70% and showed an interrelation with individual impairment levels (see Supplementary Figure S1). Consequently, using our algorithm for the wrist-only setup, performance might be more susceptive to functional impairment.

Accurate classification of body postures and physical activity types allows valuable insight into profiles of individual behavior and lays a crucial foundation for type quantification and subsequent qualitative analysis of human movement behavior. Walking and stair walking are two critical activities that define independent mobility and are associated with higher levels of physical activity and energy expenditure than static postures (Pinheiro MB et al., 2013; Creasy et al., 2016). Using the bilateral and unilateral sensor setups, walking was detected in about 87%, whereas the sensitivity for stair walking was lower. Accurately predicting walking activities by only wrist sensors remains highly challenging since hemiparetic arm swing patterns, even in standardized conditions, show great variability (Kahn et al., 2019) on one hand, and voluntary arm activity (such as grasping, transporting, and gesticulating) are present during walking in real life conditions. This mixture of walking-related and task-related upper limb movements might differ by individual ability and hence the level of functional impairment after stroke. We found an inverse relationship using only wrist sensor data, indicating higher classification performance in participants with lower self-selected walking speeds. One possible explanation for this phenomenon could be that participants with higher functional impairment were rarely able to perform upper limb tasks during gait but performed a distinguishable arm swing pattern to maintain dynamic balance. Arm swing during hemiplegic gait is highly relevant for stride synchronization (Kloter et al., 2011; Ustinova et al., 2017) and associated with increased angular motion in the mediolateral and front dorsal plane (Kahn et al., 2019), which could have generated discriminative sensor signals by wrist sensors. However, it is advisable to increase classification performance by adding at least one ankle sensor to obtain a walking-characteristic reference.

The stair walking class was generally underrepresented, and its slightly lower sensitivity might be due to the variability in stepping patterns (e.g., step-by-step and step-over-step patterns) performed by all participants. Detecting stair ascent and stair descent in participants with stroke seems to be a persisting challenge, as previous studies also reported low sensitivities, around 70%, with a full sensor configuration (Leuenberger et al., 2014). Specified mobility impairment modifies movement behavior and is highly relevant to achieving generalizability but is often underreported in activity classification studies. In some participants of our sample, impairment-associated stair walking behavior was present: low stepping speeds ascending or descending, longer pauses between steps, and holding onto the rail bilaterally. However, across sensor setups, we did not find a relationship between classification performance of stair walking and functional impairment, which might be the abovementioned variability of stepping patterns within participants.

It is a significant advantage for the discrimination between body postures when distinct sensor orientation by a key sensor location is typical for that posture. In our study, the horizontal orientation of one axis of at least one ankle and the chest sensor might have led to a sensitivity above 90% for detecting the horizontal body position while lying down. The observed slight increase of sensitivity in the classification of lying, which occurs when the chest sensor is omitted, may seem paradoxical initially. We consider the increased noise introduced by the chest due to changes in lying position or misclassification as sitting when the trunk is inclined to sitting or lying as one reasonable explanation. In our clinical experience, the application of chest sensors has been associated with discomfort and requires higher levels of patient supervision to maintain wearing compliance. Therefore, we consider it a meaningful advantage to omit the chest sensor while still maintaining excellent performance when discriminating between lying and sitting. Compared to our results, other studies in healthy adults (Horemans et al., 2020) and individuals with stroke (Fanchamps et al., 2018) have reported lower performance discriminate the two classes sitting and lying with a single thigh-mounted sensor because thigh orientation is similar in both postures. We explored misclassification incidences between postures and walking activities and found the highest misclassifications between sitting/standing and standing/walking. Although classification accuracy is high, specific information on false-positive proportions is valuable in the context of outcome interpretation. Accurate classification of physical activity types in real-life environments remains challenging because the distinction of predefined classes is impeded in natural movement containing a continuous flow of physical activities and transitions. Our results showed good performance for classifying lying, sitting, and walking, including movement transitions in a real-life environment, which is essential for further analysis and valid outcome measures.

### Clinical implications

The quantification of physical activity can be split into classification methods that categorize physical activity into discrete classes allowing for a more detailed level of physical activity and posture analysis. We introduced two classification tasks that can reliably extract specified physical activities from a continuous IMU time-series acquired from stroke patients. Both classification tasks can be applied to enable a quantitative and qualitative analysis of mobility and upper limb activities.

The first classification task broadly categorizes sensor data into walking and non-walking activities serving two purposes. Firstly, it can be used to extract walking sequences to quantify spatiotemporal gait parameters. Reduced walking speed, deviations in stride length, and stride asymmetry are characteristic of hemiplegic Gait (Viccaro et al., 2011; Mohan et al., 2021) and are associated with fall events (Moreira et al., 2015). Our highly accurate walking classification algorithm, validated under real-life conditions, might increase the reliability of step detection-based gait analysis algorithms primarily developed under laboratory-based conditions (Renggli et al., 2020; Felius et al., 2022). Secondly, our algorithm shows very low misclassification rates and can be implemented for walking exclusion to quantify upper limb movement during more static body postures. Gait-related whole-body movement was recently shown to account for 30%–40% of changes in sensor-based upper limb outcomes (Regterschot et al., 2021b). Therefore, correcting upper limb outcomes for gait-related whole-body movement is essential to increasing content validity.

Our second classification scheme branches out the classes of lying, sitting, standing, walking, and stair walking, which is opportune for two purposes. Firstly, it discriminates walking from stair walking featuring district demands on energy expenditure and energy cost in individuals with stroke (Polese et al., 2017). Since sensitivity was low for the classification of stair walking, only walking classification might be used for subsequent spatiotemporal analysis as described above. Secondly, this classification scheme allows for quantifying upper limb activity, differentiating body postures, and excluding whole-body movements. Post-stroke arm use patterns differ between sitting and standing postures (Michielsen et al., 2012), but evidence is scarce, and mechanisms remain unclear. Regterschot et al. (2021a) recently investigated longitudinal trajectories of upper limb outcome during sitting and standing, which resulted in lower change rates than studies that included whole-body movements. In the *Gait and posture* task, misclassification occurred mainly between sitting and standing but was low between static and dynamic conditions. Therefore, this algorithm can be implemented for the computation of continuous real-life measurements to analyze outcome of the time, frequency, and intensity domain. Implementation of physical activity classification algorithms is needed to expand the knowledge of motor recovery and movement behavior after stroke.

In a clinical setting, the location and number of sensors to wear might also influence patients’ compliance and wear time. Skin-mounted sensors attached to the trunk, thigh, or hip have been reported as less comfortable and are associated with increased proportions of missing data (Duncan and Murray, 2012; Troiano et al., 2014; Howie and Straker, 2016). The presented comparison of sensor configurations provides a basis for selecting a minimal yet sufficient sensing configuration for a given clinical application. We only present accuracies for classifying types of specific physical activities—sensing configurations will take into account the full analysis pipeline and the clinical end goal. For instance, although we compared bilateral and unilateral sensor configurations to classify body postures and physical activities, quantifying movement symmetry in hemiplegic individuals requires a bilateral sensor configuration.

## Limitations

This study has several limitations. Firstly, the sample was relatively small for its heterogeneity in age and motor impairment, which might have expanded the dispersion of performance metrics. A larger sample might have generated more robust results. Secondly, we included only basic physical activities in our protocol. Classification of transportation such as taking an elevator, riding by car, or public transport occurs in static body positions, although whole-body movement is detected by movement sensors which could lead to misclassification. Thirdly, we are not able to automatically predict transitions based on the IMU data. This is typically not a requirement but may be desirable in cases where transition periods need to be removed from a time-series or where the transition itself is the label of interest (Novak et al., 2013). Nevertheless, we observed that transitions in continuous data can be handled well by our method when only labels of the adjoining classes are defined to be correct.

## Conclusion

This work compares the performance of two Gait and Posture classification tasks with different levels of complexity that are ecologically validated in stroke survivors and real-life conditions. We achieved accurate classification of naturalistic body postures and walking activities performed by individuals with a wide range of motor impairment after stroke, representing a target population for the deployment of remote monitoring. This not only enables the determination of activity bout frequency, duration and intensity but also allows an in-depth analysis of movement quality. The provided classification performances across different sensor configurations enable clinicians and researchers to make informed choices using our algorithm to optimally adapt the sensor configuration to their targeted outcome of interest. The implementation of our algorithms to identify physical activity classes accurately and reliably provides a cornerstone for the research community to apply more detailed analysis algorithms to continuous data collected without contextual information. We herewith hope to contribute to the establishment of digital biomarkers derived from continuous sensing of movement with wearables for individuals with stroke.

## Data Availability

The classification algorithms of this study are available open source: https://github.com/StimuLOOP/activity-detection.
