# Supplementary material for: Accuracy of gait and posture classification using movement sensors in individuals with mobility impairment after stroke
Source: Front Physiol. 2022 Sep 26;13:933987. doi: 10.3389/fphys.2022.933987 (PMC9549863; doi:10.3389/fphys.2022.933987)
Supplement: Supplementary file 1 [file DataSheet1.PDF]

Supplementary Material

Accuracy of Gait and Posture Classification using Movement Sensors in Individuals with Mobility Impairment after Stroke

Johannes Pohl[1,2*†], Alain Ryser [3†], Janne Marieke Veerbeek[4], Geert Verheyden[2] Julia Elisabeth Vogt[3], Andreas Rüdiger Luft[1,5‡], Chris Awai Easthope[6‡]

[1]Department of Neurology, University of Zurich and University Hospital Zurich, Zurich, Switzerland

[2]Department of Rehabilitation Sciences, KU Leuven—University of Leuven, Leuven, Belgium*****

[3]Department of Computer Science, ETH Zurich, Zurich Switzerland

[4] Neurocenter, Luzerner Kantonsspital, Lucerne, Switzerland

[5]cereneo, Center for Neurology and Rehabilitation, Vitznau, Switzerland

[6]cereneo Foundation, Center for Interdisciplinary Research (CEFIR), Vitznau, Switzerland

[†] These authors share first authorship

[‡] These authors have contributed equally to this work and share last authorship

***Correspondence:**
Corresponding Author
johannes.pohl@usz.ch

Contents

1 Table S1 | Labeling definitions 3
2 Table S2 | IMU features 4
3 Table S3 | Selected hyperparameters 4
4 Table S4 | Model performance across sensor setups for *Gait Classification* with and without cross validated transitions 5
5 Table S5 | Model performance across sensor setups for *Gait and Posture Classification* including transitions in cross validation 6
6 Table S6 | Model performance across sensor setups for *Gait and Posture Classification* excluding transitions in cross validation 7
7 Performance of *Gait* classification on the individual level 8
7.1 All sensors setup 8
Figure S1 | Performance across classifiers on the individual level; acc_{bal}, balanced accuracy 8
Table S7 | Performance SVM by functional impairment (all sensors) 8
7.2 Unilateral non-affected setup 9
Figure S2 | Performance across classifiers on the individual level; acc_{bal}, balanced accuracy 9
Table S8 | Performance SVM by functional impairment (unilateral non-affected) 9
7.3 Wrists-only setup 10
Figure S3 | Performance across classifiers on the individual level; acc_{bal}, balanced accuracy 10
Table S9 | Performance SVM by functional impairment (wrists-only setup) 10
8 Performance *Gait & Posture classification* on the individual level 11
8.1 All sensors setup 11
Figure S4 | Performance across classifiers on the individual level; acc_{bal}, balanced accuracy 11
Table S10 | Performance SVM by functional impairment (all sensors) 12
8.2 Unilateral non-affected setup 13
Figure S5 | Performance across classifiers on the individual level; acc_{bal}, balanced accuracy 13
Table S11 | Performance SVM by functional impairment (unilateral non-affected setup) 14
8.3 Wrists-only setup 15
Figure S6 | Performance across classifiers on the individual level; acc_{bal}, balanced accuracy 15
Table S12 | Performance SVM by functional impairment (wrists-only setup) 16
9 Relationship classification performance vs. functional impairment across sensor setups 17

1 Table S1 | Labeling definitions

Label	Characteristics
**Lying**	**Start:** horizontal position (>45°) of trunk, lying on bed/couch. Trunk and legs have contact to surface. Legs can be bent. All position changes within horizontal orientation included (e.g., turning on back or side). **End:** Acceleration of trunk and orientation > 45° in sagittal or frontal plane
**Lying / sit**	**Start:** Acceleration of trunk and orientation > 45° in sagittal or frontal plane **End:** lying or sitting still
**Siting**	**Start:** upright or inclined position of trunk after transfer from lying or standing. Contact of buttocks to sitting surface. All trunk movement while sitting e.g.,(bending down while sitting, shoe lacing, reaching to ground) included. **End:** forward acceleration of trunk, leaving bases of support
**Sit/ stand**	**Start:** acceleration of trunk / arms to initiate movement of transfer sitting up/ down Can contain multiple attempts to stand up. **End:** standing/ sitting still or transition to other class
**Standing**	**Start:** Double support of feet, vertical trunk position. Stepping while standing maximally one step each leg while standing. Trunk movements in all plains (e.g. bending down shoe lacing or picking up object/ opening low draw. Using elevator **End**: Second consecutive stesp of one leg in any direction. Forward acceleration of trunk towards sit-to-stand transition.
**Stand/walk**	**Start:** Second consecutive step of one leg in any direction. Turning movements while standing, shuffling **End:** double support standing still or swing phase of third consecutive step
**Walking**	**Start:** third consecutive step in one direction Walking overground, including cornering, and turning when gait cycle is maintained **End:** Double support of feet, standing still
**Stairs**	**Start**: mid-swing first step ascending/descending vertical acceleration **End:** last step of stair ascend/ descend

2 Table S2 | IMU features

Domain	Feature	Accel. (post)	Accel. (act.)	Gyroscope	Altimeter
Time	Mean	✓			
	Standard Deviation	✓	✓	✓	✓
	Variance	✓	✓	✓	✓
	Inter-quartile range	✓	✓	✓	✓
	Percentile (3,10,20,97)	✓	✓	✓	✓
	Peak to peak amplitude	✓	✓	✓	✓
	Mean peak to peak amplitude	✓	✓	✓	
	Excessive kurtosis	✓	✓	✓	
	Slope				✓
	Root mean square	✓	✓	✓	✓
	Signal magnitude area		✓		
	XY correlation		✓		
	YZ correlation		✓		
	XZ correlation		✓		
Frequency	Maximal frequency component		✓		
	Energy		✓	✓	
	Entropy		✓	✓	
	Excessive kurtosis		✓		

Accel., Accelerometer; post., posture; act. Activity

3 Table S3 | Selected hyperparameters

Setup	*Gait*	*Gait & posture*
**All**	C=1	C=1
**No chest**	C=0.1	C=1
**Non-aff.**	C=1	C=1
**Affected**	C=1	C=1
**Wrists**	C=1	C=0.1

Table S4 | Model performance across sensor setups for *Gait Classification* with and without cross validated transitions

Setup	Model	Transitions excluded					Transitions included				
		Sens	Spec	Acc	Acc_{bal}	PPV	Sens	Spec	Acc	Acc_{bal}	PPV
**All**	SVM	92.6 ±6.1	92.5 ±3.8	92.5 ±3.9	92.5 ±4.7	82.4 ±13.5	90.7 ±7.5	94.5 ±4.2	93.3 ±4.3	92.6 ±5.5	85.6 ±15.3
	LR	92.1 ±6.1	93.7 ±4.8	93.0 ±4.6	92.9 ±5.1	84.5 ±16	92.1 ±6.1	93.7 ±4.8	93.1 ±4.6	92.9 ±5.1	84.5 ±16
	kNN	89.5 ±6.3	94.4 ±4.6	92.7 ±4.3	91.9 ±4.9	85.5 ±15.4	89.5 ±6.3	94.4 ±4.6	92.7 ±4.3	91.9 ±4.9	85.5 ±15.4
**No chest**	SVM	89.8 ±7.3	94.2 ±4.6	92.7 ±4.5	92.0 ±5.5	85.0 ±15.7	89.8 ±7.3	94.2 ±4.6	92.7 ±4.5	92.0 ±5.5	85.0 ±15.7
	LR	91.4 ±6.5	93.5 ±4.9	92.7 ±4.7	92.5 ±5.3	84.1 ±15.9	91.4 ±6.5	93.5 ±4.9	92.7 ±4.7	92.5 ±5.3	84.1 ±15.9
	kNN	88.5 ±6.9	94.2 ±4.5	92.3 ±4.3	91.3 ±5.3	85.0 ±15.5	88.5 ±6.9	94.2 ±4.5	92.3 ±4.3	91.3 ±5.3	85 ±15.5
**Non-aff.**	SVM	89.2 ±7.2	94.2 ±4.0	92.5 ±3.9	91.7 ±5.0	84.8 ±15.1	89.2 ±7.2	94.2 ±4	92.5 ±3.9	91.7 ±5	84.8 ±15.1
	LR	90.6 ±6.2	93.1 ±5.1	92.0 ±4.8	91.8 ±4.9	83.0 ±16.8	90.6 ±6.2	93.1 ±5.1	92.0 ±4.8	91.8 ±4.9	83.0 ±16.8
	kNN	86.9 ±6.6	94.0 ±4.2	91.5 ±4.0	90.4 ±4.9	84.4 ±15.0	86.9 ±6.6	94.0 ±4.2	91.5 ±4	90.4 ±4.9	84.4 ±15
**Aff.**	SVM	89.5 ±7.1	92.6 ±4.0	91.6 ±4.1	91.0 ±5.1	82.0 ±15.2	89.5 ±7.1	92.6 ±4	91.6 ±4.1	91.0 ±5.1	82.0 ±15.2
	LR	90.4 ±6.8	92.1 ±4.9	91.3 ±4.6	91.3 ±5.2	81.3 ±16.4	90.4 ±6.8	92.1 ±4.9	91.3 ±4.6	91.3 ±5.2	81.3 ±16.4
	kNN	86.4 ±8.1	93.7 ±4.5	91.2 ±4.3	90.0 ±5.4	83.9 ±15.5	86.4 ±8.1	93.7 ±4.5	91.2 ±4.3	90.0 ±5.4	83.9 ±15.5
**Wrists**	SVM	79.9 ±9.4	90.6 ±4.3	87.1 ±4.5	85.3 ±6.1	76.7 ±16.9	79.9 ±9.4	90.6 ±4.3	87.1 ±4.5	85.3 ±6.1	76.7 ±16.9
	LR	82.8 ±8	86.6 ±5.4	84.9 ±5.1	84.7 ±5.5	71.8 ±17.1	82.8 ±8	86.6 ±5.4	84.9 ±5.1	84.7 ±5.5	71.8 ±17.1
	kNN	80.0 ±9.1	83.8 ±4.6	82.4 ±5.1	81.9 ±5.9	67.5 ±16.4	80.0 ±9.1	83.8 ±4.6	82.4 ±5.1	81.9 ±5.9	67.5 ±16.4

Acc, accuracy; Acc_{bal}, balanced accuracy; Sens, Sensitivity; Spec, specificity; PPV, positive predictive value

5 **Table S5 | Model performance across sensor setups for *Gait and Posture Classification* <u>including</u> transitions in cross validation**

Setup	Model	Lying			Sitting			Standing			Walking			Stair walking			Overall			
		Sens	Spec	PPV	Sens	Spec	PPV	Sens	Spec	PPV	Sens	Spec	PPV	Sens	Spec	PPV	$Sens_w$	$Spec_w$	Acc	Acc_{bal}
All	SVM	93	99	86	87	96	86	87	92	84	84	95	82	75	98	85	85	95	85	86
		±15.3	±0.6	±14.5	±11.7	±3.3	±10.6	±7.1	±5.2	±11.8	±12.7	±3.5	±16.3	±5.3	±2.5	±4.4	±7.7	±2.9	±7.7	±10.2
	LR	92	100	89	83	95	84	85	91	83	81	95	82	79	97	77	83	95	83	84
		±22.4	±0.7	±15	±12.6	±3.4	±12.2	±7.3	±5.6	±12.9	±17.6	±4.3	±17.2	±7.2	±4.5	±6.3	±8.3	±2.8	±8.3	±11.2
	kNN	81	100	98	69	95	80	87	83	73	82	91	72	41	99	77	76	91	76	73
		±24.6	±0.3	±4.8	±13.3	±3.2	±11.6	±6.6	±7.2	±13.4	±12.6	±4.8	±17.1	±7.3	±1.7	±8.5	±8.9	±3.2	±8.9	±11.8
No chest	SVM	96	99	86	84	94	82	85	91	84	85	95	82	73	98	83	84	95	84	85
		±7.5	±0.7	±13.1	±11.5	±3.2	±12.8	±7.4	±6	±11.6	±12.1	±3.8	±16.7	±5.1	±2.2	±4.5	±8.4	±3	±8.4	±9.5
	LR	96	99	90	79	94	79	82	91	82	82	95	81	80	97	75	81	94	81	84
		±7.1	±1.2	±15.2	±10.7	±3.8	±15.1	±7.6	±6	±11.7	±16.2	±4.1	±17	±5.3	±4.1	±4.8	±8.4	±2.8	±8.4	±9
	kNN	75	100	98	65	93	73	84	81	71	82	91	72	39	99	69	74	89	74	70
		±24.1	±0.3	±5.5	±12.8	±3.7	±14.5	±7	±6.8	±11.6	±9.9	±4.9	±17.8	±7.5	±1	±8	±8.4	±3.1	±8.4	±11.5
Non-aff.	SVM	92	99	85	79	92	76	81	90	81	84	95	81	69	98	79	81	94	81	82
		±15.1	±0.8	±10	±10.6	±2.7	±12.6	±5.9	±5	±10.3	±14.2	±3.5	±16	±4.7	±3.2	±4.5	±7.1	±2.8	±7.1	±9.3
	LR	93	99	84	72	92	74	78	88	77	82	95	82	77	96	69	77	93	77	81
		±14.7	±0.8	±13.1	±15.9	±3.7	±14.8	±6.6	±7.4	±15	±13.5	±3.3	±15.7	±4.1	±3.3	±4.9	±7.3	±2	±7.3	±8
	kNN	75	100	96	64	90	68	79	82	71	84	91	73	42	98	68	72	89	72	70
		±21.2	±0.5	±6.6	±11.3	±3.5	±13.4	±5.9	±6.2	±12.1	±9.2	±5	±17.6	±6.8	±1.1	±5.8	±7	±2.7	±7	±10
Aff.	SVM	86	98	77	78	91	73	81	90	82	83	95	81	67	98	75	79	93	79	79
		±26.8	±1.9	±20.2	±11.4	±5.5	±15.7	±8.6	±5.2	±10.9	±10.5	±4	±16.7	±5.7	±1.3	±3.9	±7.5	±2.7	±7.5	±9.4
	LR	89	98	79	73	91	72	78	90	82	81	95	81	78	96	66	78	93	78	80
		±20.2	±2.7	±21.8	±15.1	±4.3	±16.9	±8.3	±7.1	±12.2	±11.7	±3.9	±16.8	±4.6	±1.8	±3.5	±8.4	±2.8	±8.4	±8.1
	kNN	73	100	94	63	90	68	80	81	70	80	91	71	38	98	58	71	89	71	68
		±29.3	±0.5	±11.2	±17.5	±6.1	±19.8	±9.6	±8	±12.1	±12	±4.7	±17.2	±6.4	±0.9	±6.9	±9.1	±2.9	±9.1	±11.2
Wrists	SVM	69	94	49	64	87	63	66	86	71	71	91	69	61	98	68	66	89	66	67
		±24.9	±5	±23.3	±10.3	±6.3	±19.9	±11.8	±5.1	±9.9	±14.8	±4.9	±19.7	±6.2	±1.9	±4	±9.3	±2.8	±9.3	±8
	LR	76	93	44	61	88	62	62	88	74	72	91	68	71	95	58	65	90	65	68
		±21.6	±4.2	±21.9	±9.1	±5.8	±19.6	±11.6	±4.6	±10.4	±11.7	±4.1	±18.5	±5.1	±1.8	±3.3	±7.3	±2.5	±7.3	±7
	kNN	28	98	57	61	84	57	62	82	66	74	83	56	38	98	53	60	86	60	53
		±22.1	±2.5	±30.2	±12.7	±7.2	±19.1	±11.3	±5	±10.5	±12.1	±5	±17.7	±6.8	±1	±7.7	±8.1	±2.9	±8.1	±8.3

Acc, accuracy; Acc_{bal}, balanced accuracy; kNN, k nearest neighbor, LR, logistic regression, PPV, positive predictive Sens, Sensitivity; Spec, specificity; value $Sens_w$ /$Spec_w$, weighed Sensitivity/ Specificity, SVM support vector machine

6 **Table S6 | Model performance across sensor setups for *Gait and Posture Classification* excluding transitions in cross validation**

Setup	Model	Lying			Sitting			Standing			Walking			Stair walking			Overall			
		Sens	Spec	PPV	Sens	Spec	PPV	Sens	Spec	PPV	Sens	Spec	PPV	Sens	Spec	PPV	$Sens_w$	$Spec_w$	Acc	Acc_{bal}
All	SVM	92.9	99	83.3	85.3	94	80.8	80.8	92.9	87	87.4	94.5	81.1	74.8	97.5	68.7	83.2	94.7	83.2	84.7
		±12.5	±0.8	±11.7	±10.5	±4.1	±14.9	±8	±4.6	±10.3	±10.2	±3.1	±14.5	±5.3	±1.9	±3.5	±7.1	±2.8	±7.1	±9.2
	LR	92	99.5	88.8	83.1	95.1	84	85.3	91.3	82.9	80.7	95	81.5	78.7	96.9	76.7	82.9	94.8	82.9	84.2
		±22.4	±0.7	±15	±12.6	±3.4	±12.2	±7.3	±5.6	±12.9	±17.6	±4.3	±17.2	±7.2	±4.5	±6.3	±8.3	±2.8	±8.3	±11.2
	kNN	81.3	99.9	97.8	69.2	94.5	79.7	86.8	83.4	73.2	82	90.6	71.9	41.2	98.7	77.1	76.3	90.7	76.3	73.4
		±24.6	±0.3	±4.8	±13.3	±3.2	±11.6	±6.6	±7.2	±13.4	±12.6	±4.8	±17.1	±7.3	±1.7	±8.5	±8.9	±3.2	±8.9	±11.8
No chest	SVM	95.5	99.1	85.5	84.3	94.4	81.8	84.6	91.3	83.7	84.5	95.1	82.4	72.6	98.4	83	83.7	94.5	83.7	84.8
		±7.5	±0.7	±13.1	±11.5	±3.2	±12.8	±7.4	±6	±11.6	±12.1	±3.8	±16.7	±5.1	±2.2	±4.5	±8.4	±3	±8.4	±9.5
	LR	96	99.2	89.6	79.1	93.8	78.9	82.1	90.9	82.4	82.2	94.9	81.3	80	96.7	74.5	81.2	94.3	81.2	84
		±7.1	±1.2	±15.2	±10.7	±3.8	±15.1	±7.6	±6	±11.7	±16.2	±4.1	±17	±5.3	±4.1	±4.8	±8.4	±2.8	±8.4	±9
	kNN	75.1	99.9	97.6	64.5	92.5	73.3	84.4	81.3	70.9	82.4	90.6	71.9	38.9	98.7	69	73.7	89.3	73.7	70.2
		±24.1	±0.3	±5.5	±12.8	±3.7	±14.5	±7	±6.8	±11.6	±9.9	±4.9	±17.8	±7.5	±1	±8	±8.4	±3.1	±8.4	±11.5
Non-aff.	SVM	91.8	99	85.3	79	92.2	75.7	81	90.4	81	84.2	94.7	81	69.3	97.7	78.8	80.6	93.5	80.6	81.5
		±15.1	±0.8	±10	±10.6	±2.7	±12.6	±5.9	±5	±10.3	±14.2	±3.5	±16	±4.7	±3.2	±4.5	±7.1	±2.8	±7.1	±9.3
	LR	93.4	99	84.1	71.7	92.1	73.6	78.4	87.6	76.8	81.6	95.2	81.7	76.9	96.3	68.9	77.1	93	77.1	80.5
		±14.7	±0.8	±13.1	±15.9	±3.7	±14.8	±6.6	±7.4	±15	±13.5	±3.3	±15.7	±4.1	±3.3	±4.9	±7.3	±2	±7.3	±8
	kNN	75	99.7	95.8	64.4	90.1	67.7	79.4	82.4	70.5	84.3	91.2	73.3	42.1	98.4	67.5	72.4	89.3	72.4	70.1
		±21.2	±0.5	±6.6	±11.3	±3.5	±13.4	±5.9	±6.2	±12.1	±9.2	±5	±17.6	±6.8	±1.1	±5.8	±7	±2.7	±7	±10
Aff.	SVM	85.5	98.2	77	77.5	90.6	73.1	81	90.4	81.6	82.9	94.5	80.8	67.1	98.1	75	79	92.9	79	79.4
		±26.8	±1.9	±20.2	±11.4	±5.5	±15.7	±8.6	±5.2	±10.9	±10.5	±4	±16.7	±5.7	±1.3	±3.9	±7.5	±2.7	±7.5	±9.4
	LR	88.8	98.3	79.3	73.3	91.1	71.9	78.4	90.1	81.5	80.5	94.7	80.8	78.3	96.3	66.4	77.6	93	77.6	80.1
		±20.3	±2.7	±21.8	±15.1	±4.3	±16.9	±8.3	±7.1	±12.2	±11.7	±3.9	±16.8	±4.6	±1.8	±3.5	±8.4	±2.8	±8.4	±8.1
	kNN	72.6	99.7	93.9	63.2	90	67.8	80.3	80.9	69.9	80.1	90.6	71.4	38	98.1	57.7	70.8	88.5	70.8	68.1
		±29.3	±0.5	±11.2	±17.5	±6.1	±19.8	±9.6	±8	±12.1	±12	±4.7	±17.2	±6.4	±0.9	±6.9	±9.1	±2.9	±9.1	±11.2
Wrists	SVM	68.8	93.8	48.5	64.4	87.1	63.2	66.4	85.6	71.4	71	91.3	69.2	60.8	97.5	68.1	65.9	89.4	65.9	66.5
		±24.9	±5	±23.3	±10.3	±6.3	±19.9	±11.8	±5.1	±9.9	±14.8	±4.9	±19.7	±6.2	±1.9	±4	±9.3	±2.8	±9.3	±8
	LR	75.7	93.1	43.8	61	87.6	62.1	61.6	88.2	73.6	71.5	90.8	68.3	71.3	95.4	57.6	64.8	90.3	64.8	68.1
		±21.6	±4.2	±21.9	±9.1	±5.8	±19.6	±11.6	±4.6	±10.4	±11.7	±4.1	±18.5	±5.1	±1.8	±3.3	±7.3	±2.5	±7.3	±7
	kNN	28.4	98.1	56.6	61	83.9	57	61.7	82.4	65.5	74.2	82.6	56	37.5	98.2	53.3	59.7	85.5	59.7	53.1
		±22.1	±2.5	±30.2	±12.7	±7.2	±19.1	±11.3	±5	±10.5	±12.1	±5	±17.7	±6.8	±1	±7.7	±8.1	±2.9	±8.1	±8.3

Acc, accuracy; Acc_{bal}, balanced accuracy; Sens, Sensitivity; Spec, specificity; PPV, positive predictive value

7 Performance of *Gait* classification on the individual level

7.1 All sensors setup

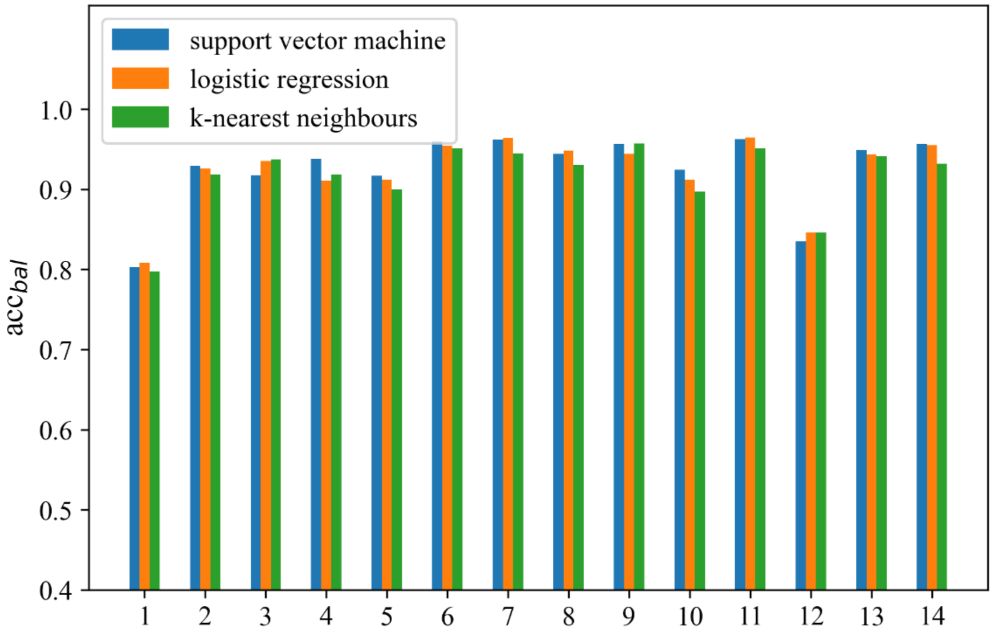

Figure S1 | Performance across classifiers on the individual level; acc_{bal}, balanced accuracy

Table S7 | Performance SVM by functional impairment (all sensors)

#	BBS	V_{10M} (m/s)	Sensitivity	Specificity	PPV	Acc	Acc_{bal}
**1**	55	1.39	76.3	84.3	43.6	83.2	80.3
**2**	45	0.39	95.4	90.5	86.0	92.4	92.9
**3**	47	0.33	91.5	92.0	85.8	91.8	91.8
**4**	52	0.92	94.4	93.2	84.6	93.6	93.8
**5**	55	0.92	93.3	90.0	78.5	91.0	91.7
**6**	49	0.63	94.2	97.9	97.0	96.3	96.0
**7**	53	0.86	97.2	95.3	92.6	96.0	96.2
**8**	56	1.08	94.3	94.6	87.9	94.5	94.4
**9**	53	0.72	100.0	91.4	71.4	92.9	95.7
**10**	35	0.25	91.6	93.3	86.5	92.8	92.5
**11**	52	1.23	97.0	95.5	90.2	96.0	96.3
**12**	48	0.79	81.1	85.8	66.6	84.6	83.5
**13**	50	0.62	95.1	94.8	86.6	94.9	94.9
**14**	49	0.59	94.4	96.9	96.1	95.7	95.6

Acc, accuracy; Acc_{bal}, balanced accuracy; Sens, Sensitivity; Spec, specificity; BBS, score Berg Balance Scale, PPV, positive predictive value, v_{10m} **;** average walking speed in m/s during 10 meter walking test

7.2 Unilateral non-affected setup

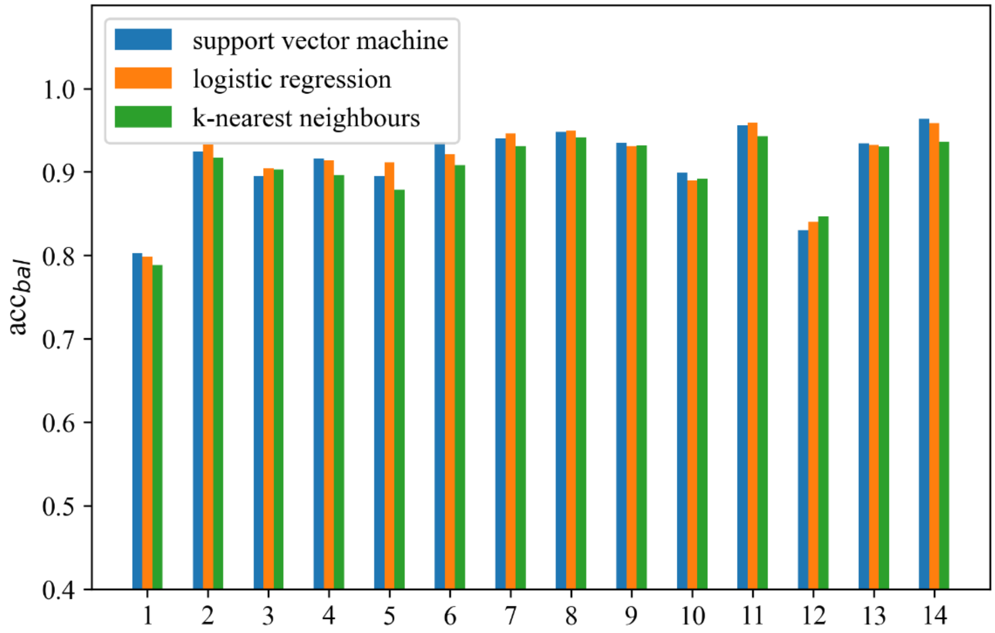

Figure S2 | Performance across classifiers on the individual level; acc_{bal}, balanced accuracy

Table S8 | Performance SVM by functional impairment (unilateral non-affected)

#	BBS	V_{10M} (m/s)	Sensitivity	Specificity	PPV	Acc	Acc_{bal}
**1**	55	1.39	74.9	81.6	38.8	80.7	78.2
**2**	45	0.39	93.3	95.0	93.3	94.2	94.1
**3**	47	0.33	86.5	94.4	89.9	91.5	90.5
**4**	52	0.92	92.5	93.2	86.9	93.0	92.8
**5**	55	0.92	89.3	87.5	76.1	88.0	88.4
**6**	49	0.63	90.3	97.7	97.2	94.3	94.0
**7**	53	0.86	92.0	94.6	90.8	93.6	93.3
**8**	56	1.08	93.2	96.9	92.4	95.9	95.1
**9**	53	0.72	98.6	90.6	64.0	91.8	94.6
**10**	35	0.25	85.2	95.4	89.9	92.1	90.3
**11**	52	1.23	96.8	96.8	92.3	96.8	96.8
**12**	48	0.79	74.0	87.9	67.6	84.4	80.9
**13**	50	0.62	93.6	95.6	87.5	95.1	94.6
**14**	49	0.59	94.6	98.7	98.6	96.7	96.7

Acc, accuracy; Acc_{bal}, balanced accuracy; Sens, Sensitivity; Spec, specificity; BBS, score Berg Balance Scale, PPV, positive predictive value, v_{10m} **;** average walking speed in m/s during 10 meter walking test

7.3 Wrists-only setup

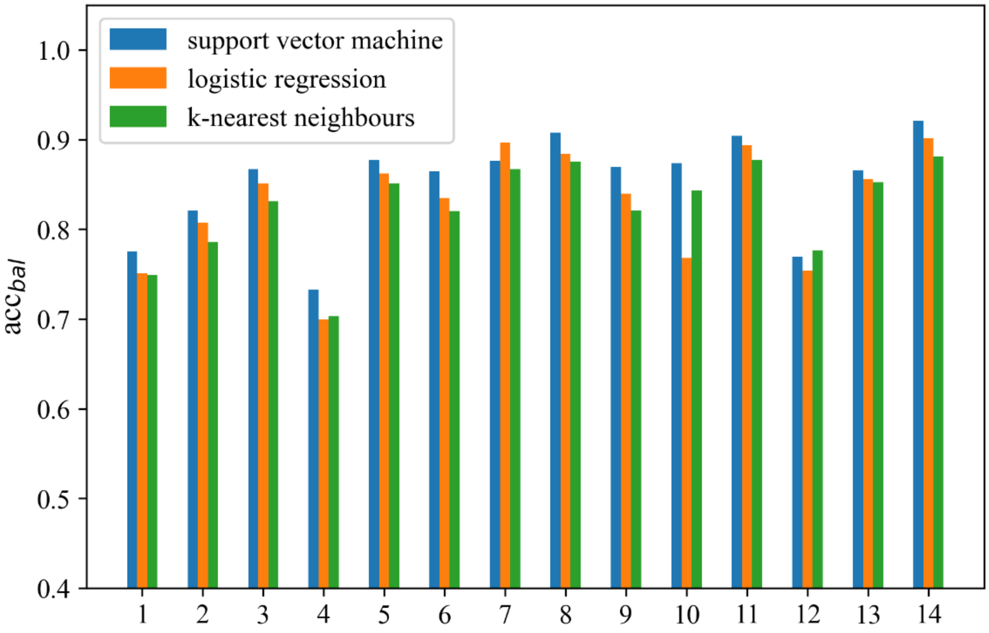

Figure S3 | Performance across classifiers on the individual level; acc_{bal}, balanced accuracy

Table S9 | Performance SVM by functional impairment (wrists-only setup)

#	BBS	V_{10M} (m/s)	Sensitivity	Specificity	PPV	Acc	Acc_{bal}
**1**	55	1.39	74.9	81.6	38.8	80.7	78.2
**2**	45	0.39	93.3	95.0	93.3	94.2	94.1
**3**	47	0.33	86.5	94.4	89.9	91.5	90.5
**4**	52	0.92	92.5	93.2	86.9	93.0	92.8
**5**	55	0.92	89.3	87.5	76.1	88.0	88.4
**6**	49	0.63	90.3	97.7	97.2	94.3	94.0
**7**	53	0.86	92.0	94.6	90.8	93.6	93.3
**8**	56	1.08	93.2	96.9	92.4	95.9	95.1
**9**	53	0.72	98.6	90.6	64.0	91.8	94.6
**10**	35	0.25	85.2	95.4	89.9	92.1	90.3
**11**	52	1.23	96.8	96.8	92.3	96.8	96.8
**12**	48	0.79	74.0	87.9	67.6	84.4	80.9
**13**	50	0.62	93.6	95.6	87.5	95.1	94.6
**14**	49	0.59	94.6	98.7	98.6	96.7	96.7

Acc, accuracy; Acc_{bal}, balanced accuracy; Sens, Sensitivity; Spec, specificity; BBS, score Berg Balance Scale, PPV, positive predictive value, v_{10m} ; average walking speed in m/s during 10 meter walking test

8 Performance *Gait & Posture classification* on the individual level

8.1 All sensors setup

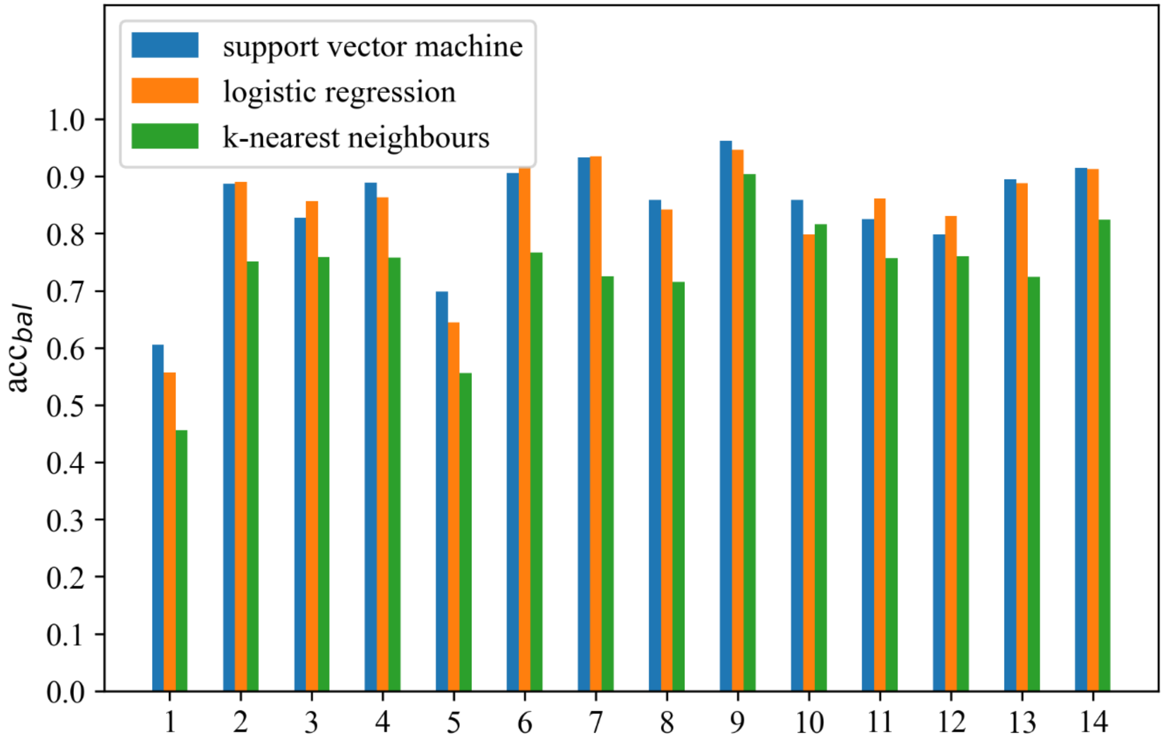

Figure S4 | Performance across classifiers on the individual level; acc_{bal}, balanced accuracy

Table S10 | Performance SVM by functional impairment (all sensors)

#	BBS	V_{10M} (m/s)	LYING Sens	LYING Spec	LYING Acc_{bal}	SITTING Sens	SITTING Spec	SITTING Acc_{bal}	STANDING Sens	STANDING Spec.	STANDING Acc_{bal}	WALKING Sens	WALKING Spec	WALKING Acc_{bal}	STAIRS Sens	STAIRS Spec	STAIRS Acc_{bal}	OA Acc_{bal}
**1**	55	1.39	83.5	93.0	88.3	60.7	91.4	76.0	59.6	82.6	71.1	66.2	86.6	76.4	16.2	97.2	56.7	57.2
**2**	45	0.39	100.0	97.5	98.7	88.3	88.8	88.5	65.7	96.6	81.1	92.1	94.0	93.0	90.7	97.5	94.1	87.4
**3**	47	0.33	82.1	94.5	88.3	73.5	91.1	82.3	70.9	94.0	82.5	93.8	94.6	94.2	74.6	97.7	86.2	79.0
**4**	52	0.92	100.0	95.8	97.9	85.7	86.9	86.3	66.3	96.3	81.3	83.7	95.5	89.6	89.7	95.4	92.6	85.1
**5**	55	0.92	73.2	96.1	84.7	74.6	89.3	82.0	64.4	90.9	77.6	91.5	89.0	90.3	42.6	97.3	70.0	69.3
**6**	49	0.63	100.0	96.8	98.4	91.4	95.6	93.5	84.5	96.9	90.7	93.2	95.9	94.6	72.5	99.6	86.1	88.3
**7**	53	0.86	99.3	96.9	98.1	88.7	97.3	93.0	81.1	96.7	88.9	92.4	97.2	94.8	97.9	98.1	98.0	91.9
**8**	56	1.08	100.0	96.0	98.0	91.2	91.1	91.1	70.4	97.4	83.9	90.3	92.5	91.4	61.1	96.0	78.5	82.6
**9**	53	0.72	100.0	99.1	99.5	94.4	98.8	96.6	88.0	100.0	94.0	99.0	95.9	97.5	0.0*	97.5	48.7	95.4
**10**	35	0.25	100.0	93.6	96.8	80.0	99.6	89.8	86.3	94.7	90.5	53.8	96.1	75.0	0.0*	87.3	43.6	80.0
**11**	52	1.23	99.1	96.1	97.6	80.1	98.1	89.1	79.6	93.9	86.7	76.9	96.8	86.9	96.6	94.5	95.5	86.4
**12**	48	0.79	100.0	95.8	97.9	63.2	92.0	77.6	74.6	88.6	81.6	77.5	90.0	83.8	74.6	98.0	86.3	78.0
**13**	50	0.62	99.2	97.4	98.3	61.8	96.7	79.2	83.2	82.4	82.8	94.8	96.7	95.8	84.7	98.2	91.5	84.8
**14**	49	0.59	100.0	95.6	97.8	75.6	98.7	87.1	86.1	92.6	89.3	91.0	98.8	94.9	90.0	97.3	93.7	88.5

Acc, accuracy; Acc_{bal}, balanced accuracy; Sens, Sensitivity; Spec, specificity; BBS, score Berg Balance Scale (/56), OA, overall classes, v_{10m} **;** average walking speed in m/s during 10 meter walking test; * these participants did not perform stair walking activity

8.2 Unilateral non-affected setup

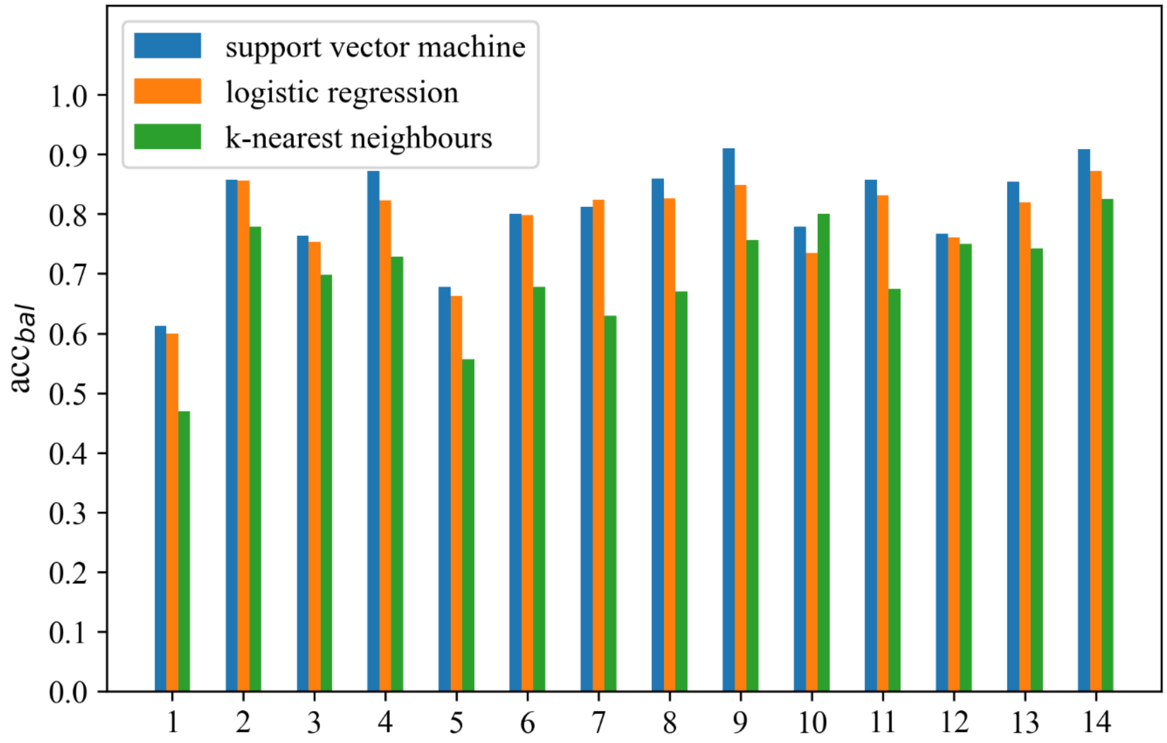

Figure S5 | Performance across classifiers on the individual level; acc_{bal}, balanced accuracy

Table S11 | Performance SVM by functional impairment (unilateral non-affected setup)

	BBS	V_{10M}	LYING			SITTING			STANDING			WALKING			STAIRS			OA
**#**		(m/s)	**Sens**	**Spec**	**Acc_{bal}**	**Sens**	**Spec**	**Acc_{bal}**	**Sens**	**Spec.**	**Acc_{bal}**	**Sens**	**Spec**	**Acc_{bal}**	**Sens**	**Spec**	**Acc_{bal}**	**Acc_{bal}**
**1**	55	1.39	84.2	97.8	91.0	60.2	93.4	76.8	67.6	81.5	74.6	61.5	86.6	74.1	24.3	94.7	59.5	59.6
**2**	45	0.39	100.0	98.5	99.2	86.2	89.5	87.8	72.2	94.6	83.4	89.0	95.6	92.3	82.6	98.5	90.5	86.0
**3**	47	0.33	100.0	97.0	98.5	75.4	89.7	82.5	73.9	88.5	81.2	89.9	96.3	93.1	57.9	97.7	77.8	79.4
**4**	52	0.92	100.0	98.0	99.0	77.6	90.6	84.1	77.1	92.9	85.0	83.0	96.0	89.5	82.1	96.6	89.3	83.9
**5**	55	0.92	47.2	96.2	71.7	53.5	90.2	71.8	72.5	84.3	78.4	86.9	86.5	86.7	36.1	96.4	66.2	59.2
**6**	49	0.63	100.0	97.3	98.7	61.6	90.7	76.2	74.3	89.2	81.7	91.0	96.3	93.6	74.4	99.4	86.9	80.3
**7**	53	0.86	97.6	94.2	95.9	57.1	93.1	75.1	69.1	90.6	79.9	88.7	95.3	92.0	85.6	98.3	92.0	79.6
**8**	56	1.08	100.0	97.5	98.7	87.7	90.4	89.1	78.3	95.4	86.8	87.7	93.0	90.4	66.4	99.3	82.8	84.0
**9**	53	0.72	90.9	98.1	94.5	81.4	92.7	87.1	81.1	98.7	89.9	98.6	96.2	97.4	0.0*	95.0	47.5	88.0
**10**	35	0.25	100.0	98.3	99.1	71.8	96.3	84.1	87.1	83.9	85.5	35.7	95.9	65.8	0.0*	84.0	42.0	73.7
**11**	52	1.23	98.0	96.8	97.4	88.0	96.0	92.0	78.9	96.4	87.7	82.9	97.4	90.2	89.7	96.9	93.3	87.5
**12**	48	0.79	100.0	97.7	98.9	50.0	88.1	69.1	72.8	79.6	76.2	68.4	91.7	80.1	71.6	97.4	84.5	72.6
**13**	50	0.62	97.2	95.9	96.6	59.5	93.1	76.3	82.1	82.4	82.2	91.9	97.2	94.6	80.6	99.0	89.8	82.3
**14**	49	0.59	100.0	97.0	98.5	76.8	94.7	85.7	78.2	91.2	84.7	88.6	98.5	93.5	94.0	97.8	95.9	87.5

Acc, accuracy; Acc_{bal}, balanced accuracy; Sens, Sensitivity; Spec, specificity; BBS, score Berg Balance Scale (/56), OA, overall classes, v_{10m} **;** average walking speed in m/s during 10 meter walking test; * these participants did not perform stair walking activity

8.3 Wrists-only setup

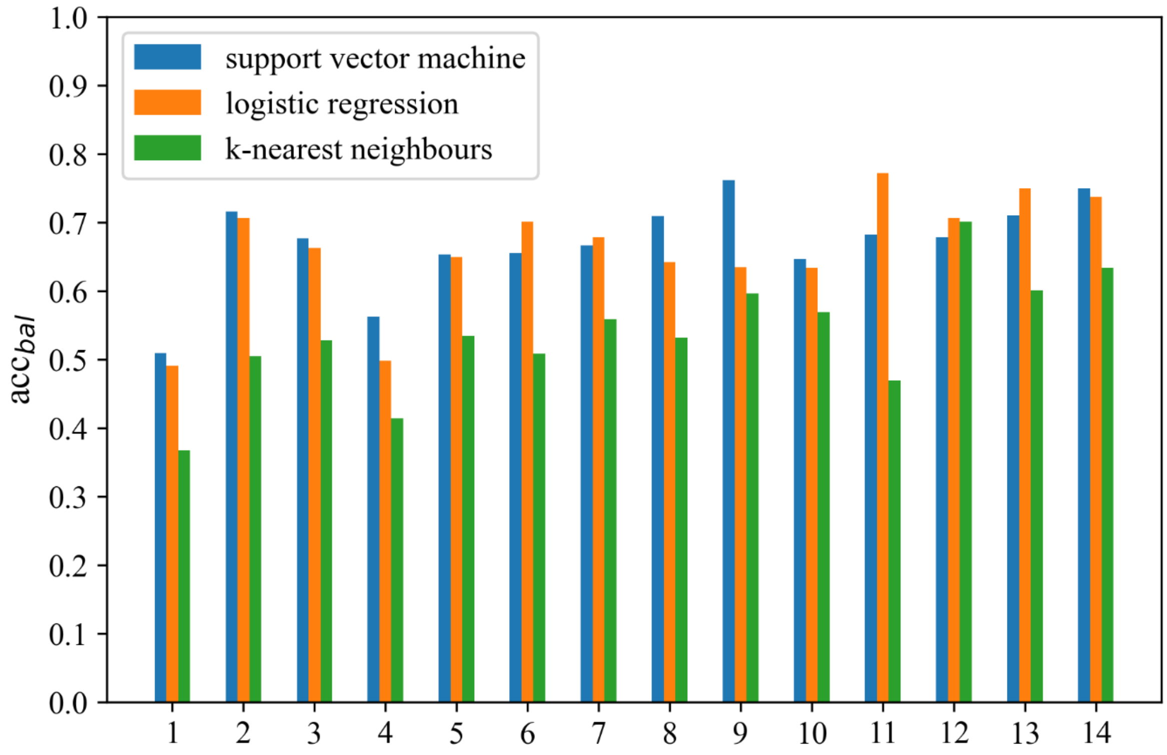

Figure S6 | Performance across classifiers on the individual level; acc_{bal}, balanced accuracy

Table S12 | Performance SVM by functional impairment (wrists-only setup)

	BBS	V_{10M}	LYING			SITTING			STANDING			WALKING			STAIRS			OA
#		(m/s)	Sens	Spec	Acc_{bal}	Sens	Spec	Acc_{bal}	Sens	Spec.	Acc_{bal}	Sens	Spec	Acc_{bal}	Sens	Spec	Acc_{bal}	Acc_{bal}
**1**	55	1.39	52.7	93.5	73.1	57.7	81.6	69.7	50.4	84.8	67.6	54.6	80.1	67.4	32.4	98.2	65.3	49.6
**2**	45	0.39	95.8	88.9	92.4	57.2	87.6	72.4	57.8	84.9	71.4	63.4	93.4	78.4	79.1	97.6	88.3	70.7
**3**	47	0.33	96.0	86.4	91.2	47.7	90.7	69.2	54.3	86.8	70.5	82.5	94.2	88.4	78.6	96.0	87.3	71.8
**4**	52	0.92	44.4	83.4	63.9	49.3	78.4	63.8	43.8	79.3	61.5	29.3	93.1	61.2	89.7	93.6	91.7	51.3
**5**	55	0.92	100.0	87.8	93.9	48.8	96.5	72.6	68.1	84.1	76.1	82.4	86.5	84.4	31.1	98.7	64.9	66.1
**6**	49	0.63	86.2	93.0	89.6	74.1	84.5	79.3	60.1	91.4	75.7	79.2	94.4	86.8	60.6	99.4	80.0	72.0
**7**	53	0.86	56.8	91.3	74.1	66.9	83.5	75.2	51.3	86.9	69.1	74.4	95.0	84.7	87.6	98.9	93.3	67.4
**8**	56	1.08	100.0	84.9	92.5	60.4	82.1	71.3	50.0	94.1	72.0	77.9	83.8	80.8	19.5	99.7	59.6	61.6
**9**	53	0.72	86.4	83.8	85.1	62.1	91.1	76.6	57.6	92.7	75.2	76.4	89.7	83.0	0.0*	97.0	48.5	70.6
**10**	35	0.25	69.4	86.9	78.2	58.8	97.2	78.0	77.9	89.5	83.7	82.1	93.5	87.8	0.0*	98.3	49.2	72.1
**11**	52	1.23	62.4	95.2	78.8	87.1	86.2	86.7	55.6	96.0	75.8	65.1	92.7	78.9	84.5	93.9	89.2	71.0
**12**	48	0.79	100.0	91.5	95.7	58.3	90.6	74.5	68.3	85.4	76.9	60.2	87.9	74.0	55.2	98.5	76.8	68.4
**13**	50	0.62	93.5	95.1	94.3	54.4	96.2	75.3	76.0	85.4	80.7	77.3	88.7	83.0	77.6	95.5	86.5	75.8
**14**	49	0.59	93.8	91.0	92.4	70.3	95.2	82.8	65.4	88.5	77.0	82.9	96.9	89.9	82.0	98.1	90.0	78.9

Acc, accuracy; Acc_{bal}, balanced accuracy; Sens, Sensitivity; Spec, specificity; BBS, score Berg Balance Scale (/56), OA, overall classes, v_{10m} **;** average walking speed in m/s during 10 meter walking test; * these participants did not perform stair walking activity

9 Relationship classification performance vs. functional impairment across sensor setups

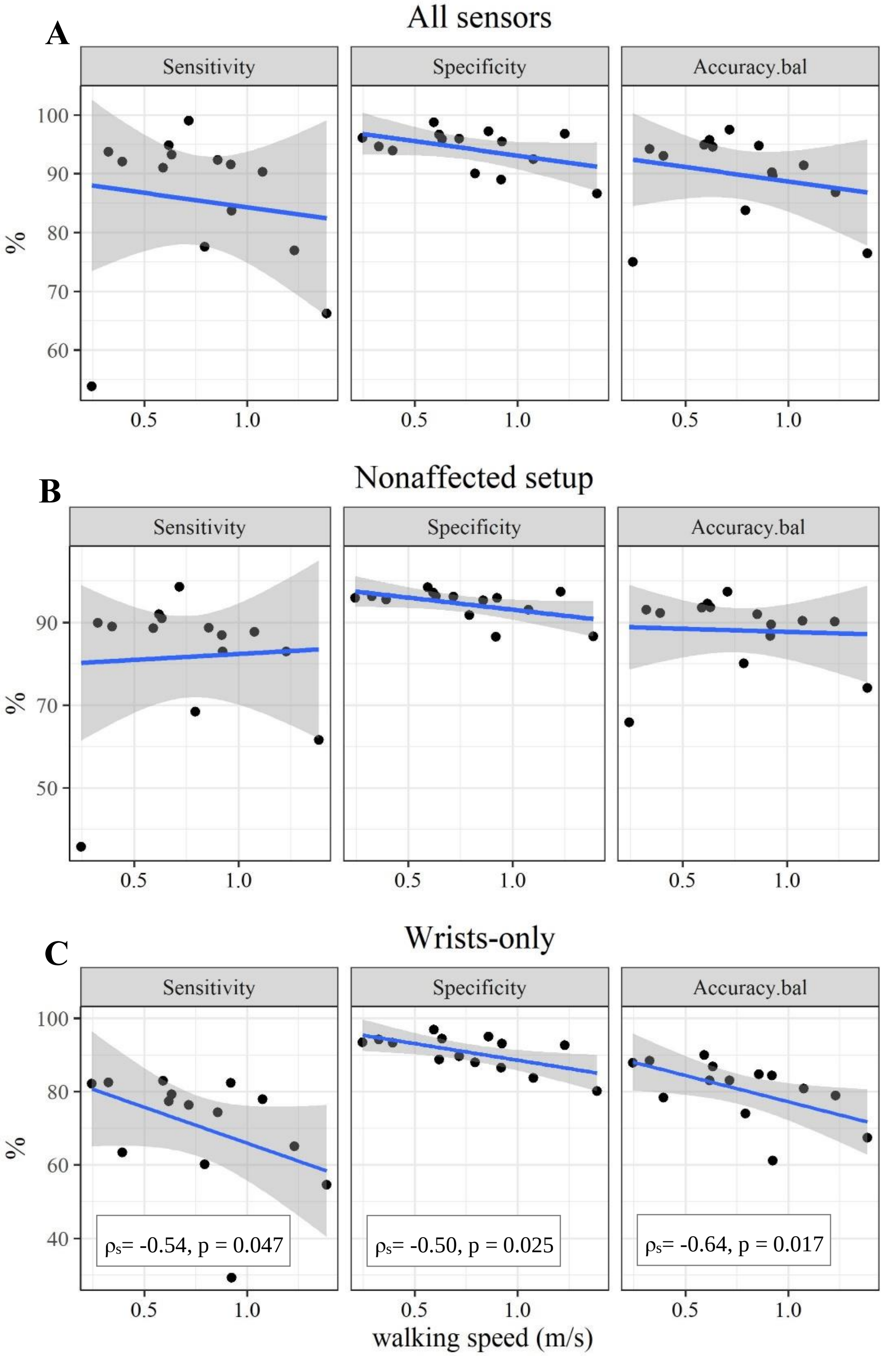

Figure S7 | Relation between classification performance walking and 10m-meter walking speed for sensor configurations: All sensors, bilateral setup (**A**), nonaffected ankle and wrist sensor, unilateral setup (**B**), and the wrists-only setup (**C**), Spearman correlation coefficient ρ_s and p-value is shown upon significant relationships ($p<0.05$).
